# Simplification of Mobility Tests and Data Processing to Increase Applicability of Wearable Sensors as Diagnostic Tools for Parkinson’s Disease

**DOI:** 10.3390/s24154983

**Published:** 2024-08-01

**Authors:** Rana M. Khalil, Lisa M. Shulman, Ann L. Gruber-Baldini, Sunita Shakya, Rebecca Fenderson, Maxwell Van Hoven, Jeffrey M. Hausdorff, Rainer von Coelln, Michael P. Cummings

**Affiliations:** 1 Center for Bioinformatics and Computational Biology, University of Maryland, College Park, MD 20742, USA; rmkhalil@umd.edu; 2 Department of Neurology, University of Maryland School of Medicine, Baltimore, MD 21201, USA; lshulman@som.umaryland.edu (L.M.S.); rfenders12@gmail.com (R.F.); mvanhoven2@gmail.com (M.V.H.); 3 Department of Epidemiology and Public Health, University of Maryland School of Medicine, Baltimore, MD 21201, USA; abaldin@som.umaryland.edu (A.L.G.-B.); sshakya@som.umaryland.edu (S.S.); 4 Center for the Study of Movement, Cognition, and Mobility, Neurological Institute, Tel Aviv Medical Center, Tel Aviv 6492416, Israel; jeffh@tlvmc.gov.il; 5 Department of Physical Therapy, Faculty of Medicine & Health Sciences, Tel Aviv University, Tel Aviv 6997801, Israel; 6 Sagol School of Neuroscience, Tel Aviv University, Tel Aviv 6997801, Israel; 7 Rush Alzheimer’s Disease Center, Rush University Medical Center, Chicago, IL 60612, USA; 8 Department of Orthopedic Surgery, Rush University Medical Center, Chicago, IL 60612, USA

**Keywords:** Parkinson’s disease, wearable sensors, machine learning, mobility testing, diagnosis, movement disorders, movement analysis

## Abstract

Quantitative mobility analysis using wearable sensors, while promising as a diagnostic tool for Parkinson’s disease (PD), is not commonly applied in clinical settings. Major obstacles include uncertainty regarding the best protocol for instrumented mobility testing and subsequent data processing, as well as the added workload and complexity of this multi-step process. To simplify sensor-based mobility testing in diagnosing PD, we analyzed data from 262 PD participants and 50 controls performing several motor tasks wearing a sensor on their lower back containing a triaxial accelerometer and a triaxial gyroscope. Using ensembles of heterogeneous machine learning models incorporating a range of classifiers trained on a set of sensor features, we show that our models effectively differentiate between participants with PD and controls, both for mixed-stage PD (92.6% accuracy) and a group selected for mild PD only (89.4% accuracy). Omitting algorithmic segmentation of complex mobility tasks decreased the diagnostic accuracy of our models, as did the inclusion of kinesiological features. Feature importance analysis revealed that Timed Up and Go (TUG) tasks to contribute the highest-yield predictive features, with only minor decreases in accuracy for models based on cognitive TUG as a single mobility task. Our machine learning approach facilitates major simplification of instrumented mobility testing without compromising predictive performance.

## 1. Introduction

Parkinson’s disease (PD) is the most common neurodegenerative movement disorder and affects an estimated 7–10 million people worldwide [1]. It is characterized by a range of motor symptoms, including tremors [2,3], bradykinesia [4,5], rigidity [4,6], a slow shuffling gait with small steps [7,8,9], freezing of gait [10,11], and imbalance [7,12]. Among all these motor symptoms, gait impairment and imbalance most substantially impact the quality of life of PD patients, ultimately resulting in disability and loss of independence [13,14].

Currently, PD diagnosis is based on clinical assessment, including history and neurological examination, which is inherently subjective in nature and depends on the level of experience of the healthcare provider. Consequently, initial clinical diagnosis by movement disorders experts has limited accuracy (79.6%, increasing to 83.9% at follow-up) and limited overall validity [15]. The need for objective, low-cost, reliable, and valid biomarkers that can accurately identify patients with PD was identified as one of the top priorities in PD research by a National Institutes of Health/National Institute of Neurological Disorders and Stroke (NIH/NINDS) panel [16].

Wearable sensors containing accelerometers and gyroscopes show great promise in objectively detecting the presence of key PD motor symptoms in a convenient and accessible manner [17,18,19,20,21]. The widespread availability of wearable sensors makes it possible to quantitatively assess mobility without costly equipment or a dedicated gait laboratory [22]. Used in instrumented mobility testing, these sensors provide highly granular data on gait, balance, and other motor functions to identify features and patterns indicative of PD [23,24]. Ultimately, the expectation is for wearable sensors to support earlier detection and treatment of PD, leading to better outcomes for patients [18,25]. However, critical challenges have yet to be overcome in order to turn sensors from research instruments into clinical tools widely used to support the diagnosis of PD in clinical practice [26,27].

Studies on the use of wearable sensors to diagnose PD vary widely in terms of which and how many sensors to use and where to place them [28,29,30,31,32,33,34,35]. There is also a large variation in which and how many instrumented mobility tasks participants are required to perform [29,36,37,38]. Moreover, the methods employed to process sensor data and derive relevant features are complex and vastly different across studies [29,39,40,41,42].

**Figure 1:**
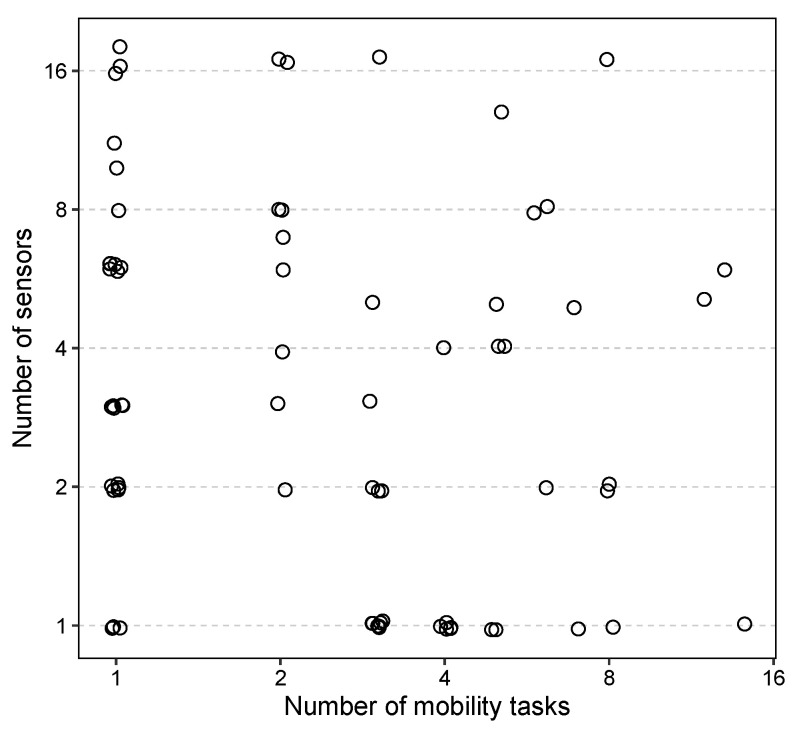
Number of mobility tasks and sensors used in prior studies. The axes are in log_2_ scale.

We conducted a search of the literature from 2017 to 2024 to identify previous studies that used wearable sensors for PD assessment. This involved reviewing three prior review articles [33,34,35] and performing a search of the PubMed database with the keywords “(wearable OR IMU) AND Parkinson’’, which yielded 72 relevant articles. Our survey revealed that the number of sensors used in these studies ranged from 1 to 18, and the number of tasks ranged from 1 to 14, occurring in various combinations (Figure 1). We categorized these studies into four groups based on their objectives. The first group included studies focusing on PD diagnosis. There were 21 studies in this group, using between 1 and 18 sensors and from 1 to 12 tasks [43,44,45,46,47,48,49,50,51,52,53,54,55,56,57,58,59,60,61,62,63]. Among these, six studies used a single sensor placed on the lower trunk [48,56,59], foot [49], wrist [54], or torso [60]. Additionally, 11 studies used data from a single mobility task—either simple gait tasks [43,46,51,53,57,59,61,62,63] or more complex tasks [52,56]—with only two of these studies also using a single sensor [56,59]. The second group, comprising 24 studies, focused on quantifying/monitoring the severity of PD symptoms [30,64,65,66,67,68,69,70,71,72,73,74,75,76,77,78,79,80,81,82,83,84,85,86], with 4 using a single sensor [64,65,66,67] and 7 using a single mobility task [66,68,69,70,71,72,73]. Only one study in this group used both a single sensor and a single mobility task [66]. The third group, consisting of 15 studies, aimed to detect freezing of gait in PD [87,88,89,90,91,92,93,94,95,96,97,98,99,100,101], with 8 using a single sensor [87,88,89,90,91,92,98,99] and 2 using a single mobility task [93,94]. The last group included 12 studies that performed quantitative characterization of gait or identified imbalance in PD [32,102,103,104,105,106,107,108,109,110,111,112], with 3 using a single sensor [102,103,104] and 5 using a single mobility task [32,105,106,107,112]. None of the studies from the third or fourth groups used both a single sensor and a single mobility task. The detailed results of the surveyed studies are presented in Supplementary Table S1.

The reviewed papers exhibit a variety of approaches using wearable sensors for PD assessment, focusing on different tasks and parameters. Some studies emphasize the importance of comprehensive data collection to train symptom detection models [85], objectively measure gait parameters [43,46,48,50,51,52,69,86,96,102,103,106,112], monitor daily life activities [70,96], or detect early stages of the disease [32,48,57,62,100]. The choice of the number of sensors and their placement is often driven by the specific objectives of each study. For instance, capturing hand motor tests [44,64,78], assessing balance impairments [32,47,66,86,108], and measuring dyskinesia severity [77] require specific sensor setups. Sensors are commonly placed on the wrist for tremor and bradykinesia monitoring [54,64,68,74,76,78,80,83,99]; on the foot or shoe to measure gait parameters and detect freezing gait and gait phases [43,49,53,69,88,97,105,106]; on the waist, hips, torso, or lower back for general gait and balance analysis [48,50,59,60,90,91,98,104]; and on the lower limbs to measure several key aspects of PD [61,82,94,101,111,112]. Some studies integrate multiple sensor locations to capture a broader range of symptoms, such as axial rigidity and postural instability, providing comprehensive data [30,45,55,62,63,70,72,73,75,79,96,110]. Overall, the reviewed studies aim to balance detailed data capture with practical application, ensuring accurate and relevant information for their specific goals.

Increasing the number of tasks (abscissa of Figure 1) is associated with more time required from both the patient and clinician, while an increasing number of sensors (ordinate) indicates greater complexity in terms of maintaining, storing, and placing sensors, as well as processing the data. Therefore, the goal of this project is rigorous simplification of the number of sensors, mobility testing, and data processing protocols based on a systematic and data-driven analysis of comprehensive sensor-based mobility data.

Machine learning offers a powerful and flexible analytical approach to accomplish this goal in a large-scale, high-dimensional, and complex dataset collected by wearable sensors [31,113,114,115,116]. Furthermore, machine learning can identify and capture hidden patterns and relationships that would be hard to detect using standard observation and statistical methods [117].

Although previous studies have successfully used machine learning and wearable sensors for PD diagnosis [45,49,50,52,53,55,57,59,60,61,62,63], wearable sensors are still not commonly applied in clinical settings due to the complexity and variability of proposed protocols for mobility testing and data processing. Our study objective is to employ machine learning techniques to identify strategies that streamline and simplify the process, reducing the time and effort required by both participants and clinicians while maintaining the predictive performance of models to diagnose (or support the diagnosis of) PD based on instrumented mobility testing. This will ease the application of wearable sensors as a diagnostic tool in clinical practice.

As an initial step toward the simplification of mobility testing, we used a single sensor positioned on the lower backs of participants. Then, we show that sensor data collected from participants performing a single mobility task in a conventional clinic hallway can effectively differentiate individuals with PD from controls. Moreover, we demonstrate that manual segmentation and complex calculations of kinesiological features, which have been prevalent in prior studies [30,36,38,39,118,119], may be unnecessary. Instead, algorithmic segmentation, followed by the calculation of a small set of sensor-derived features from a single sensor and a single mobility task, offers substantial simplification of both mobility testing and data processing without significantly compromising predictive accuracy. Feature importance analysis revealed that timed up and go (TUG) tasks, which include walking, turning, and sit-to-stand and stand-to-sit transitions, provided more valuable information to our models than simpler mobility tasks such as gait and sway assessments. These results suggest that substantial simplification in mobility testing with a wearable sensor and subsequent data analysis is possible without loss of diagnostic accuracy for PD, facilitating the integration of wearable sensors into clinical practice.

## 2. Materials and Methods

### 2.1. Participants

Our study on sensor-based mobility analysis in parkinsonism (“Towards Next-Generation Phenotyping in Parkinson Disease: Quantitative Analysis of Gait and Balance Using a Portable Biosensor Device”) at the University of Maryland Movement and Memory Disorders Center (UM-MMDC) enrolled a total of 368 participants between October 2015 and March 2020, including 50 control participants and 318 participants with parkinsonism. Of those, 293 were diagnosed with idiopathic PD by a UM-MMDC movement disorder specialist, applying the UK Parkinson’s Disease Society Brain Bank Clinical Diagnostic Criteria [120]. All participants with parkinsonism were also enrolled in the Health Outcomes Measurement (HOME) study at UM-MMDC. The HOME study is a naturalistic cohort study that collects patient- and clinician-reported data during routine office visits. In addition to HOME study participation, all 318 participants with parkinsonism plus 50 non-parkinsonian age-matched control participants were separately enrolled in the mobility analysis in the parkinsonism study. Patients were excluded from the mobility analysis study if they were unable to stand or walk without assistance (Hoehn & Yahr stage 5); presented with other conditions unrelated to parkinsonism that, in the judgment of the treating physician, significantly interfere with gait and balance (e.g., advanced lower extremity arthritis or severe lower extremity neuropathy); or failed to provide signed consent. A total of 24 of the study participants had previously undergone deep brain stimulation (DBS) surgery (20 to the subthalamic nucleus (STN), 3 to the globus pallidus internus (GPi), and 1 to the thalamic ventral intermediate (Vim) nucleus). All participants signed informed consent. A person accompanying a patient (spouse/significant other, sibling, or friend) was approached for enrollment as a control participant if they were of similar age to the patient and did not carry a diagnosis of parkinsonism or another condition significantly affecting their gait and balance. All study procedures for both the HOME study and the mobility analysis study were approved by the University of Maryland Institutional Review Board.

Of 318 participants with parkinsonism, 293 were diagnosed with idiopathic PD. The remaining 25 participants were diagnosed with other forms of parkinsonism. Participants with non-PD forms of parkinsonism were excluded from data analysis for this study. Of 293 participants with a diagnosis of idiopathic PD, one was unable to complete the study procedures and was excluded. Of the remaining 292 PD participants, 30 were excluded from our analysis due to missing data (2 due to technical problems with the wearable sensor and 28 due to a lack of important clinical data). None of the 50 control participants were excluded.

### 2.2. Clinical Evaluations

Participants were enrolled during routine outpatient visits at the UM-MMDC. All PD participants underwent full clinical assessment according to the HOME study protocol, as previously described [13,121,122,123]. In brief, collected general data included basic demographics, Cumulative Illness Rating Scale-Geriatrics (CIRS-G) [124], the Older Americans Resource and Services (OARS) Disability Subscale [125] (activities of daily living (ADLs) and instrumental activities of daily living (IADLs)), a questionnaire on the use of assistive devices and falls (ADF), PROMIS Profile 29 [126], and the Montreal Cognitive Assessment (MoCA) [127]. PD-specific data included PD duration (time since symptom onset and time since diagnosis), PD severity (Total and Motor (Part III) Unified Parkinson’s Disease Rating Scale (UPDRS) [128], and modified H&Y staging [14,129]). The HOME study is a naturalistic study, and patients are enrolled during routine clinic visits and on their regular medications. For 87% of assessments performed for this study, the patients rated themselves as being in a medication ON state. Patients who had previously undergone DBS surgery were examined in the stimulation ON state, both for clinical assessments and for the instrumented mobility testing.

### 2.3. Assessment of Mobility

Mobility testing with a wearable sensor was performed using a previously published protocol [38,130,131]. We adopted the mobility testing protocol from the Memory and Aging Project and the Minority Aging Research Study at the RUSH Alzheimer’s Disease Center at Rush University [132] based on the previous successful use of mobility data from this study in parkinsonism. In brief, participants performed mobility tasks with a single wearable sensor strapped to their lower back. For this study, the following data from five of those mobility tasks were analyzed: 1. a 32-foot walk, where participants walk eight feet back and forth twice without stopping, including three turns; 2. standing with eyes open, where participants stand with their feet shoulder-width apart for 20 s with their eyes open; 3. standing with eyes closed, where participants stand with their feet shoulder-width apart for 20 s with eyes closed; 4. two trials of the timed up and go test (TUG), where participants stand up from a seated position in a chair, walk eight feet to a line on the floor, turn, walk back to the chair, make a second turn, and sit down again; and 5. two trials of cognitive TUG (cogTUG), where participants perform the TUG test while counting backwards from 100 in steps of 3.

### 2.4. Device and Data Collection

Preceding the mobility testing procedure, a small (106.6 × 58 × 11.5 mm) lightweight (55 g) sensor device (Dynaport MT, McRoberts B.V., The Hague, The Netherlands, technical specifications: https://www.mcroberts.nl/products/movetest/, accessed on 17 June 2024) was strapped to the participant’s lower back with a neoprene belt around the waist. This device contains a triaxial accelerometer (range: ± 8 g; resolution: 1 mg) and a triaxial gyroscope (range: ±2000 dps; resolution: 70 dps) with 100 Hz sampling of three-dimensional acceleration (vertical (az), mediolateral (ay), and anteroposterior (ax)) and rotation (yaw (gz), pitch (gy), and roll (gx)) of the lower trunk. Recording by the sensor was controlled (started and stopped) remotely via wireless Bluetooth signals from a notebook computer using a handheld controller. To facilitate post hoc segmentation of individual mobility tasks, digital markers were placed to label the beginning and end of each mobility task. At the end of each test session, the sensor was removed from the participant and connected to an encrypted notebook computer for data transfer. Raw data files were then transferred to a secure data server.

### 2.5. Data Extraction

A custom-made, runtime-based Matlab graphic user interface (GUI) was used to view the accelerometry data. The 32-foot walk, TUG, and cogTUG were identified by the typical accelerometry pattern of each of these tasks and by the digital markers recorded at the time of mobility testing. The two 20 s segments corresponding to standing with eyes open and closed, respectively, were identified solely based on the digital markers recorded at the time of mobility testing. The raw accelerometry data for each individual mobility task were saved as separate files for subsequent data processing.

**Figure 2:**
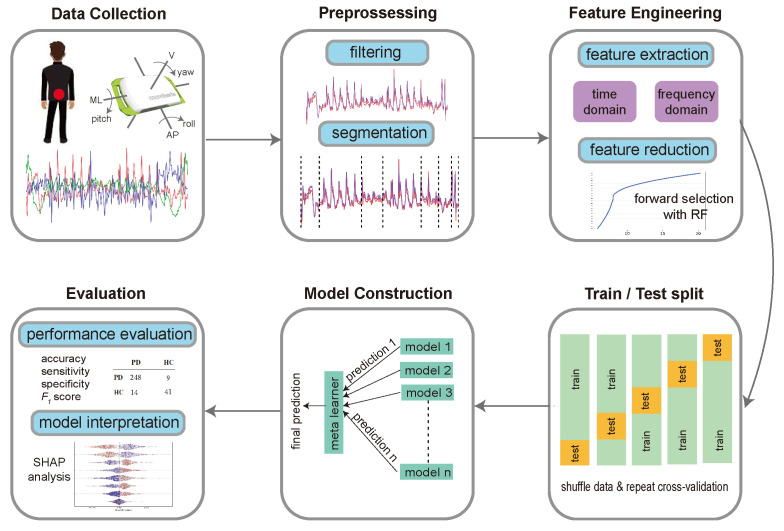
Machine learning pipeline. The process started by collecting data from a wearable sensor on three linear and three angular axes. Filtration was then applied to reduce noise in signals using a Butterworth filter. The composite tasks were further processed by dividing them into segments corresponding to the different subtasks. Using the six signals recorded for each participant and task, a large set of time-domain and frequency-domain features was calculated. This set was reduced using forward selection based on feature importance calculated with random forests. The data were then shuffled multiple times, and a cross-validation framework was applied in each iteration to generate train and test sets. A super-learner model was built using a wide array of base models, and the final prediction was calculated by assigning a weight to the prediction of each base learner. Finally, the performance of the model was evaluated using accuracy, sensitivity, specificity, and F_1_ score measures. Model interpretation was performed using SHapley Additive exPlanation (SHAP) analysis (see Supplementary Methods) to quantify the contributions of features to the final model prediction. AP: anteroposterior direction; V: vertical direction; ML: mediolateral direction.

Figure 2 presents an overview of the machine learning pipeline implemented in this study. The following sections describe the steps of the workflow in detail.

### 2.6. Segmentation

Some motor tasks performed in this study are composed of several subtasks. For instance, the TUG and cogTUG tasks consist of the following six components: sit to stand, two turns, two walks, and stand to sit. The 32-foot walk task has seven parts representing the four walks and three turns. We developed a segmentation procedure extending a previously described algorithm [133] to include more tasks and subtasks. Our algorithm starts by excluding the stationary segments at the start and end of the signal. These stationary segments result if the recording starts before the participant starts the task or the recording stops after the participant terminates the task. The largest constant parts (with small slope change) common among the six channels and found at the start or the end of the signal were defined as stationary segments and were trimmed. Then, the turns of a task were detected by calculating the angular position from the angular velocity ib the anteroposterior axis (gx) using the trapezoidal integration method [134]. The model for turning fits a line with three horizontal segments and two segments with constant slopes. The transition points from zero to constant and from constant to zero slopes were used to mark the starts and ends of the turns (Supplementary Figure S1a). This method of determining the onset and offset time points of 180° turns has been previously validated [135]. The same integration method was applied to the acceleration data in the vertical direction (az) to determine the sit-to-stand and stand-to-sit parts of the TUG and cogTUG tasks. Specifically, the start and the end of the peak of the fitted line represent the sit-to-stand part, and the start and the end of the valley represents the stand-to-sit part (Supplementary Figure S1b). Finally, our algorithm marked the segments not mapped as turns in the 32-foot walk task and those between the sit-to-stand part and the first turn or between two turns in the TUG and cogTUG tasks as walking segments. Supplementary Figures S2 and S3 show the different components extracted for a participant performing the TUG and 32-foot walk tasks, respectively. Once we applied our segmentation procedure, the time-domain and frequency-domain features described in the next section were calculated separately for each subtask.

### 2.7. Feature Engineering

For each of the six channels recorded for each participant and mobility test, we started by applying a low-pass, 20 Hz, zero-lag, 4th-order Butterworth filter [136] to reduce noise in signals. Then, we derived the following sets of features from each signal: statistical measures consisting of 23 frequency-domain features (Supplementary Table S2); 44 time-domain features (Supplementary Table S3); the top-ten power amplitudes from Discrete Fourier Transform (DFT) and Lomb–Scargle Periodogram (LSP) [137,138]; and three time-domain features calculated across the signals of each pair of the six channels (Supplementary Table S4). These sensor-derived features were combined from several previous studies [29,42,139,140,141]. Kinesiological features were calculated/extracted using a second custom-made, runtime-based, mobility task-specific Matlab graphic user interface (GUI) as previously described [40,41,142]. Additionally, we included features not derived from the sensor data, including demographics and other variables (e.g., age, race, gender, height, OARS, and ADF scores) as predictive variables. Each participant was represented using a total of 26,515 features calculated from the six channels of the segmented subtasks of the 32-foot walk, standing-with-closed-eyes task, standing-with-open-eyes task, the segmented tasks of the TUG task (from the first trial and the second trial and the mean of corresponding features from both trials), and the segmented subtasks of the cogTUG task (from the first trial and the second trial and the mean of corresponding features from both trials). Preprocessing steps were applied to the data before running machine learning models, including removal of variables with constant or infinite values, imputation of missing values using the median of observed values, and normalization of each variable by subtracting its mean and dividing it by its standard deviation.

### 2.8. Feature Selection

To reduce the number of features, we applied forward feature selection. The goal was to select an optimal subset of features that results in a simpler model with similar or better performance. We started by ranking all features by their importance using a random forest model after randomly undersampling the larger group. Then, the elbow point of the feature importance plot was defined as the point that is farthest away from a straight line drawn from the first to the last point of the plot. This elbow point was used as a cut-off threshold to select the set of candidate features. If the size of this set was $n_{thr}$, then multiple models were trained using the top one, top two, or …, top $n_{thr}$ important features. The best model was selected as the one that minimizes the total Akaike information criterion (AIC) [143], and the corresponding set of features was used in subsequent analyses. This variable reduction strategy results in fewer features compared to the use of principal component analysis, as reported in similar studies [38,144,145], and retains the original features.

### 2.9. Machine Learning Model

Prior to model training, we split the data into five groups. We used stratified sampling to preserve the class frequencies within each group. Then, a nested-loop framework was applied, where in each iteration of the inner loop, a five-fold cross-validation was implemented by choosing four groups for training and one for testing of the model. This ensured that each participant was included exactly once in the test data. The benefit of this technique is that all observations were used for both training and testing. In the outer loop, we created five replicates by randomly shuffling the whole dataset using different random number generator seeds to create different combinations of participants within each training and testing group. The final class of each participant was defined as the majority vote of the predicted classes from the five outer loops.

Prior to constructing each classifier, a balanced sampling process was applied to the training sets, where the mean sample sizes of the two classes were calculated and used to undersample the majority class and/or oversample the minority class. This mean-based sampling approach provides a balance between simplicity and computational efficiency. An ensemble method combining multiple machine learning algorithms was then applied to the training sets. This approach is known to achieve performance equal to or better than any of the base learners. The user of a super learner (SL) [146] was proposed as a stacked generalization using an ensemble of methods that generates an optimal model by creating a weighted linear convex combination of a set of base algorithms. The super-learner algorithm compares the performance of the user-chosen base algorithms by applying *k*-fold cross-validation to determine the weighted contribution of each base algorithm to the ensemble. We used the R interface H2O with five-fold cross-validation and a generalized linear model as a meta learner. For base models, we included a generalized linear model (GLM) [147], distributed random forests (DRFs) [148], gradient boosting machines (GBMs) [149], extreme gradient boosting (XGBoost) [150], and neural networks [151]. Hyperparameters of the base algorithms were selected using grid search criteria. The final ensemble incorporated 327 base models, including 11 GLM models, 100 DRF models, 100 GBM models, 16 XGBoost models, and 100 neural network models (see Supplementary Files). The class prediction thresholds were selected based on the highest F_1_ score.

Given the predicted class of each participant, the performance of the classification model was assessed using overall accuracy, area under the receiver operating characteristic curve (AUC-ROC), sensitivity, specificity, and F_1_ score.

### 2.10. Group Feature Importance

We employed the concept of group feature importance to assess the information of various mobility tasks in predicting the outcome of interest. Group feature importance refers to the assessment of the collective information of a set of features within a specific category. All features derived from each mobility task were grouped together to form five separate groups corresponding to the five mobility tasks. We also created an additional group by combining features not derived from the sensor data, including demographics and other variables (e.g., age, race, gender, height, OARS, and ADF scores). To measure group importance, we adapted the permutation importance method for feature groups within the context of random forests, building upon a previously outlined approach [152]. For each feature group, the grouped variables were simultaneously permuted, and the resultant mean decrease in accuracy prior to and post permutation was computed.

### 2.11. Statistical Analysis

Data analysis was performed with R version 4.2.3 (2023-03-15) and RStudio. A two-tailed Student’s *t*-test was applied to compare continuous variables, and Pearson’s $\chi^{2}$ test was used to compare categorical variables between two groups. Results are presented as mean ± s.d. for continuous variables. Actual *p* values for the statistical tests are reported.

For each performance metric of the models, we display both the computed values and their respective 95% two-sided confidence intervals (CIs). These intervals were derived via balanced bootstrap resampling with 10K replicates and were adjusted using bias correction and acceleration to better manage bias and skewness in the bootstrap distribution. The bootstrap replicates were generated using the boot function in R package boot version 1.3–30 [153,154], and the boot.ci function from the same package was used to generate the CIs.

## 3. Results

We collected wearable sensor data from 262 participants with PD (87% were in a medication ON state) and 50 age-matched controls. We partitioned PD participants by their modified Hoehn & Yahr (H&Y) stage, defining mild PD as H&Y ≤ 2, moderate PD as H&Y 2.5 and 3, and severe PD as H&Y 4 (note that H&Y 5 patients were excluded from this study). Although our analysis encompasses four classifiers to distinguish control participants from all PD, mild PD, moderate PD, and severe PD groups, respectively, here, we focus on the results of our models for all PD participants and mild PD participants only. The all-PD classifier best mimics a routine clinical situation with patients in various stages of PD. The mild-PD classifier focuses on the group of PD participants that is most similar to control participants, making these two groups the most challenging to differentiate from one another. Results of the models for moderate- and severe-PD participants are detailed in the Supplementary Results (Section S2.1). Table 1 provides the characteristics of the study cohort.

After the participants performed the five motor tasks (Section 2.3) wearing a Dynaport MT sensor strapped to their lower back, we followed the experimental design described in Figure 2 to predict the final class of each participant.

### 3.1. Classification of PD versus Control

By applying the forward feature selection approach, the total number of features was reduced from 25,705 to 9 for the all-PD classification and 14 for the mild-PD classification (Supplementary Figures S4–S6). Eight of the selected features for the all-PD classification were derived from one or both trials of the cogTUG task; three features were from the second cogTUG trial (cogTUG2), one feature was from the first cogTUG trial (cogTUG1), and four features were the mean of the corresponding features from the two cogTUG trials (mean cogTUG). One feature was derived from the open-eyes standing task. For the mild-PD classification, 13 of the 14 selected features were derived from the cogTUG task; 2 features were from cogTUG2, 1 feature was from cogTUG1, and 10 were from mean cogTUG. One feature was from the eyes-closed standing task. The selected cogTUG features for both classifiers were derived from the turn, walk, and stand-to-sit components of the task, representing acceleration and rotational measures along the vertical, mediolateral, and anteroposterior axes. None of the selected features for both classifiers were from the 32-foot walk or TUG tasks.

Among the 327 base models included in the ensemble classifier, a gradient boosting machine model made the greatest weighted contribution to the final models (Supplementary Figures S7 and S8). The models trained on the selected features achieved an overall accuracy of 92.6% (confidence interval [CI] 88.8%, 94.9%) in distinguishing all-PD from control participants and 89.4% (CI 84.3%, 92.3%) in distinguishing those with mild PD from control participants. Confusion matrices (prediction summaries) and performance metrics for the classifiers are presented in Table 2 and Table 3, respectively.

**Figure 3:**
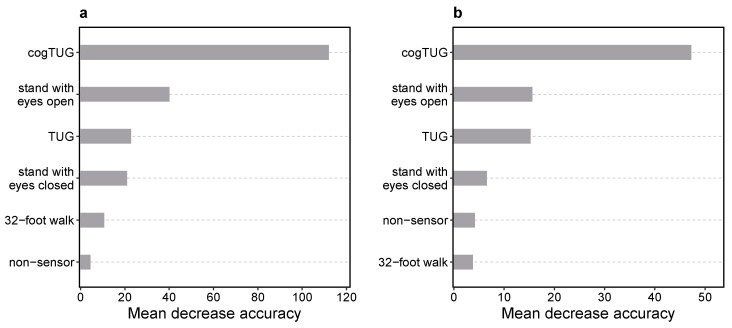
Group feature importance of models distinguishing controls from (**a**) all Parkinson’s disease (PD) and (**b**) mild-PD (H&Y ≤ 2) participants. Groups are ranked by their importance calculated by permuting features within each group simultaneously and reporting the mean decrease in accuracy between the original and permuted data. Under the null hypothesis that there is no association between the group of predictor variables and the model prediction, permutation should have no or little impact on predictive performance. More detailed individual feature-level importance scores and Shapley additive explanation (SHAP) values are illustrated in Supplementary Figures S4 and S6.

To further elucidate which mobility tasks were most relevant for the performance of our models, we conducted a group feature importance analysis (see Section 2.10). Figure 3 illustrates the group feature importance of the five mobility tasks and the non-sensor (demographic and clinical) features. The highest-yield group was the one with cogTUG-derived features for both models distinguishing controls from all-PD and mild-PD participants. A substantial decline of at least 64% in importance was observed when comparing the cogTUG group with the remaining groups for either model, underscoring the remarkable discriminatory power inherent in the cogTUG features. More detailed individual feature-level importance scores and Shapley additive explanation (SHAP) values were also calculated (Supplementary Figures S4 and S6, Supplementary Methods, Supplementary Results (Section S2.2)).

To identify potentially confounding factors that may impact our models, we conducted a comparison of demographic and clinical characteristics between correctly classified and misclassified PD and control participants. A two-tailed Student’s *t*-test was applied to compare continuous variables, and Pearson’s $\chi^{2}$ test was used to compare categorical variables between separate groups. The comparison results are summarized in Table 4. For both classifiers (all PD and mild PD), false positives (FPs, i.e., controls falsely classified as PD) were significantly older compared to the correctly classified controls (true negatives, TNs). False negatives (FNs, i.e., PD participants classified as controls) were younger compared to the correctly classified PD participants (true positives, TPs), but this difference was not statistically significant. These results suggest that older controls have movement characteristics that confuse the classifier, but there was no significant difference in age between the correctly and falsely classified PD participants. Also, the mean Unified Parkinson’s Disease Rating Scale part III (UPDRS_PIII) motor score of the FNs (falsely classified PD participants) was significantly lower than that of TPs (correctly classified PD participants). In contrast to the UPDRS_PIII scores, misclassified PD participants were evenly distributed among the different H&Y stages. Similarly, there was no significant difference in the medication state, sex, MoCA scores, or CIRS-G scores between the correctly and falsely classified PD participants, suggesting that these factors do not have a confounding impact on model performance.

### 3.2. Strategies for Simplifying Mobility Testing and Its Associated Workload

One of the major obstacles preventing widespread use of wearable sensors for PD diagnosis is the increased burden in terms of time and effort in performing sensor-based mobility testing and subsequent data processing and analysis. To overcome these obstacles, we first focused on possible simplification of the data analysis process. As detailed above, our data processing pipeline includes an algorithmic segmentation approach (Section 2.6) to calculate a set of sensor-derived frequency-domain and time-domain features from the resulting task segments, referred to as “standard models” below. To evaluate the effectiveness of the automated segmentation method and whether segmentation is necessary at all, we constructed alternative models using features derived from data of the unsegmented tasks. These models decreased the accuracy in distinguishing PD from control participants by 3.2% and 5.1% for all-PD and mild-PD classifications, respectively, compared to our standard models (Table 5, second column vs. first column).

We also developed another set of models incorporating calculated kinesiological features (see Section 2.7), in addition to the time- and frequency-domain features from the algorithmic segmentation tasks. These models also reduced the accuracy by 4.8% and 0.50% compared to our standard models (Table 5, third column vs. first column). These findings show that algorithmic task segmentation increases diagnostic accuracy, but complex calculation of kinesiological features does not. These time-consuming and labor-intensive additional steps required to calculate the kinesiological features can therefore be eliminated without compromising the predictive performance of our diagnostic models.

As a second step towards simplification of the overall workflow, we focused on the tasks of mobility testing itself, by assessing the predictive performance achievable with fewer mobility tasks. Given the outsized importance of cogTUG features in the context of our models based on data from all available mobility tasks (Figure 3), we examined the performance of two drastically simplified models, using TUG- or cogTUG-derived features only, respectively, in addition to the non-sensor (demographic and clinical) features. We applied the feature selection approach to these single-task models (see Supplementary Table S5 for the selected features). For the all-PD classifier, the TUG-only model and cogTUG-only model decreased the accuracy by 2.5% and 3.2%, respectively (Table 6, columns 2 and 3, row 1). For participants with mild PD, applying the cogTUG-only model decreased the accuracy by 2.4%, whereas the accuracy dropped more substantially (by 11.7%) using the TUG-only model compared to the standard model based on data from all tasks (Table 6, columns 2 and 3, row 2). These results suggest that sensor data from a single appropriately chosen mobility task may be sufficient to achieve satisfactory performance in differentiating PD and control participants. Specifically, a dual task paradigm (cognitive plus mobility task) like the cogTUG task achieves superior diagnostic performance to a mobility task alone (like the TUG task) in identifying participants in the early stages of PD.

Our observation that data from additional mobility tasks only offer a limited increase in diagnostic accuracy led us to consider whether further radical simplification would be possible without compromising the performance of our models. Therefore, we tested whether the total duration of TUG or cogTUG tasks as a single feature may be sufficient to build classifiers with predictive performance similar to that of our more comprehensive models described above. Even though we found a significant time difference between PD and control participants for both TUG and cogTUG tasks (see Supplementary Results, Section S2.3), the corresponding logistic regression models using total duration as a single predictor variable decreased the accuracy in distinguishing PD from control participants by at least 8.6% compared to our standard models (Table 6, columns 4 and 5 compared to column 1). Based on these results, we conclude that total TUG/cogTUG duration alone is not sufficient to capitalize on the discriminatory potential of these complex tasks.

## 4. Discussion

Our study demonstrates the effectiveness of analyzing wearable sensor data to distinguish PD participants from controls using the proposed machine learning pipeline for five mobility tasks. By leveraging a large dataset comprising participants in different stages of PD and controls performing five mobility tasks, along with a carefully selected subset of sensor-derived features and suitable machine learning models, we achieved an accuracy of 92.6% in identifying participants across different PD stages, surpassing the accuracy of 81% previously reported for clinical diagnosis by movement disorder experts [15]. We focused on our modeling results for all-PD participants and for mild-PD participants specifically. The all-PD classifier represents a typical clinical scenario with patients at various stages, while the mild-PD classifier targets the group most similar to control participants, where differentiation is most challenging [155] and where wearable sensors could be most clinically relevant.

Previously published studies have reported accuracies ranging from 73.5% to 96% in discriminating between PD and control participants [29,36,37,39,45,52,55,59,61,63,115,119,156]. Some studies have utilized the AUC-ROC as a performance measure, with values ranging from 0.86 to 0.99 [50,113,157], compared to an AUC of 0.86 for our model. Additional studies have reported sensitivity and specificity metrics, with sensitives ranging from 40% to 96.2% and specificity ranging from 82% to 96.9% [28,49,60,158], as compared to 95% sensitivity and 82% specificity in our case. Major differences between studies in terms of the number of participants, PD severity, tasks conducted, types and quantity of sensors employed, and reported performance metrics likely account for these differences in diagnostic performance and make direct comparison very challenging.

Analysis of group and individual feature importance revealed that the most important mobility tasks for classifying PD participants are the TUG and cogTUG tasks (Figure 3). Both tasks involve various everyday activities, including walking, turning, and sit-to-stand and stand-to-sit transitions. Our results demonstrate that more complex mobility tasks with multiple mobility subtasks provide diagnostically superior information compared to simpler motor tasks. Models based on the cogTUG task as a classic cognitive–motor dual task, in particular, showed superior performance in differentiating between participants with mild PD and controls (see Table 6). Simpler tasks, such as standing with eyes open, standing with eyes closed, and the 32-foot walk were much less important. Previous studies have reported a strong correlation between these simpler motor assessments and PD [30,159,160,161,162]. In the context of our study and with the included mobility tasks and features, we demonstrate that simpler motor tasks are not as informative as more complex ones and, thus, can be omitted from the battery of our mobility tests with very minor loss of predictive performance. This is consistent with the results of a previous study that utilized multiple sensors and a higher number of sensor-derived features [163]. However, our results show that further simplification is possible using a single sensor and a reduced set of features, even with a single mobility task.

In our machine learning framework, we implemented automatic segmentation of the composite tasks into their subtask components, coupled with the calculation of many sensor-derived features combined from prior studies. Segmentation of complex tasks into subtasks (e.g., sit to stand, walk, and turn for the TUG and cogTUG tasks) increased the diagnostic accuracy of our models compared to models based on unsegmented data (see Table 5). This automatic segmentation, as opposed to the use of manual annotations in other studies [39,118], not only expedites the processing but also minimizes subjective biases as a source of errors. Additionally, we employed ensembles comprising disparate machine learning classifiers. This approach distinguishes our study from others that primarily depend on individual models [30,32,39,119,156,158], thereby improving predictive accuracy and robustness. Weighted ensembles have demonstrated performance equal to or better than that of any constituent individual learner [146,164], as they leverage the diverse properties and strengths of various algorithms. Although we observed a modest performance difference of approximately 1% between the ensembles and the models with the highest weight in each ensemble, the process of finding the optimal model requires searching through multiple models and their respective hyperparameters.

Many kinesiological variables can be calculated based on signal processing and analysis of accelerometric and gyroscopic data from a wearable sensor using a developed software. This provides quantitative movement characterization in kinesiological terms, as demonstrated in previous studies [38,43,119,165]. However, these variables require additional manual input for the developed software, and our analysis demonstrates that these kinesiological features are neither necessary nor more helpful than analysis based on sensor-derived features alone in achieving better model performance (see Table 5). This observation suggests that properties of movement beyond those corresponding to conventional kinesiological variables were captured in our feature set derived from data from a wearable sensor and that those properties were instrumental in elevating the predictive performance of our models. Eliminating the additional data processing steps required for the generation of kinesiological variables significantly reduces the workload associated with sensor-based mobility testing and facilitates the use of quantitative mobility testing in research (e.g., testing large numbers of participants) and clinical practice.

Finally, we attempted radical simplification of our models by examining the time to complete either the TUG or cogTUG task as the only feature in our models. Total task duration was significantly shorter for controls compared to PD participants (see Supplementary Results, Section S2.3). However, using elapsed time alone as the basis for discriminating PD was markedly less accurate than models including additional sensor-derived features (see Table 6). Another study also showed moderate accuracy (AUC = 0.699) when discriminating between PD patients and controls using the total duration of a TUG test performed at a self-selected speed [166]. The decrease in model performance demonstrates a trade-off between simplicity of testing and data analysis on the one hand and predictive accuracy (and, hence, the value of mobility testing) on the other hand. It is worth noting that if a model based on total duration alone performed equally as well as our multi-feature-based models, one would conclude that timing the TUG (or cogTUG) task with a stopwatch is just as good as using a state-of-the-art wearable sensor. Our results show that is not, in fact, the case. Rather, our models clearly show the added benefit of using a highly granular mobility analysis provided by a wearable accelerometer/gyroscope.

We observed that increasing the diversity of sensor-derived features improves diagnostic accuracy. Our use of forward feature selection greatly reduced the number of features included in the model. Consequently, it is not the number of features per se but the nature of the features that determines the accuracy. Including multiple tasks gives us some additional informative features, as does segmenting tasks into subtasks. On the whole, the latter appears to be the best approach, as it requires reduced time and effort for both patients and clinicians, as the additional effort is handled algorithmically by a computer.

One of the strengths of our study is the large sample size and the wide spectrum of PD severity of our PD participants. We included 262 participants with PD and 50 control participants, compared to an *n* of 13 to 20 in many other previously published studies [28,32,167]. Our PD participants differed widely in terms of disease severity, ranging from mild (H&Y ≤ 2) to moderate (H&Y 2.5 and 3) and severe (H&Y 4). Therefore, participants in this study represent the full spectrum of ambulatory PD patients encountered in clinical practice, compared to previous studies that focused on a more limited range of disease severity [28,32,167]. Another strength is the simplicity of instrumented mobility testing using a single wearable sensor. This is in contrast to the multiple sensors employed in many other studies [30,32,36,116] and facilitates clinical use and scalability by streamlining data collection and analysis.

Although our proposed set of features might not be easily interpretable by clinicians, it shows potential for distinguishing between PD and control participants. Given our primary aim of achieving the highest possible diagnostic accuracy and real-world applicability, it is essential to include features that contribute to this goal, regardless of their interpretability. Studies that emphasize interpretability might consider using features that are more clinically explainable and may apply prior correlation analysis, as feature importance measures can show bias towards correlated predictor variables [168,169].

In addition to typical motor symptoms, PD is characterized by non-motor features including cognitive impairment and autonomic dysfunction (e.g., dizziness due to a drop in blood pressure in an upright position). Such non-motor features were not assessed in control participants in our study and, therefore, could not be included in our machine learning models, which is one of the limitations of our study. Incorporating such non-motor manifestations of PD might lead to even more accurate models to predict (or identify) PD participants and will be part of our future work. Additionally, while the H&Y scale is commonly used to assess overall PD severity, it is limited by its inter-rater variability due to its reliance on clinical judgment. This subjectivity introduces potential inconsistencies in the partitioning of PD participants, raising the question of how appropriate the H&Y scale is as frame of reference for objective assessment of PD severity. Information regarding the levodopa equivalent daily dose (LEDD) of each PD participant is not available in the PD clinical database (HOME study) we used for this study. However, ON/OFF medication status, which was available, was not found to be an important feature in any of our machine learning analyses, which is evidence that LEDD and ON/OFF status are not consequential in discrimination of PD patients and controls.

Objective and granular measures of disease manifestation as provided by a wearable sensor are promising candidates as superior markers of disease severity and disease progression. However, longitudinal studies are necessary to test this hypothesis. The cross-sectional nature of the data presented here is another limitation of our study. Longitudinal analysis of a subgroup of our study cohort is currently ongoing. Another aspect worth exploring in more detail in future studies is the potential impact of confounding factors on classification performance. Our analysis of misclassified participants shows that certain variables that are likely to be associated with the level of medical comorbidity, such as age, could influence the sensor data and classification outcomes. Older controls were falsely classified as PD, and PD participants with relatively minor motor symptoms (low UPDRS_PIII motor score) were falsely classified as controls. The differentiation between only mildly affected PD patients and older (non-PD) adults is also among the biggest challenges in clinical routine, which is why, for clinicians, the use of a wearable sensor as a supportive biomarker is relevant and attractive in such situations. That was also the rationale for us focusing on models for mild PD versus controls, in addition to all PD versus controls, in this project. Future studies with an even larger sample size for those particular groups of participants may facilitate the development of more comprehensive models that incorporate movement characteristics to overcome these limitations of our models and to further enhance the accuracy and reliability of sensor-based PD classification/identification.

## 5. Conclusions

In conclusion, our study demonstrates that diagnostic accuracy for PD exceeding that typically achieved by movement disorders experts in a routine clinical setting is possible with few or even just one complex mobility task instrumented with a single wearable sensor when appropriately analyzed using machine learning. Our results provide a framework for such simplification and the ultimate harmonization of existing approaches to facilitate the use of wearable sensors and machine learning-based PD diagnosis in clinical settings.

## Figures and Tables

**Table 1:** Characteristics of the study cohort.

Feature	Controls (n=50)	All PD (n=262)	Mild PD (n=185)
Age (years, $\bar{x}\pm SD$)	64.1 ± 9.8	66.9 ± 9.3	65.4 ± 9.1
Gender (%male)	38.0	62.0	58.4
Height (cm, $\bar{x}\pm SD$)	168.1 ± 10.9	172.2± 10.4	172.0 ± 10.3
UPDRS (total, $\bar{x}\pm SD$)	–	35.5 ± 17.1	29.4 ± 13.4
UPDRS (motor-part III, $\bar{x}\pm SD$)	–	22.2 ± 11.8	18.5± 9.7
Disease duration (years, $\bar{x}\pm SD$)	–	7.8 ± 6.5	6.6 ± 5.6
H&Y ($\bar{x}\pm SD$)	–	2.2 ± 0.62	1.9 ± 0.26
stage 1 (n)	–	12	12
stage 1.5 (n)	–	4	4
stage 2 (n)	–	169	169
stage 2.5 (n)	–	35	–
stage 3 (n)	–	25	–
stage 4 (n)	–	17	–

**Table 2:** Confusion matrices of the classifiers using controls and participants with Parkinson’s disease (PD). Rows represent actual classes, and columns represent predictions.

(a) Controls vs. All PD			(b) Controls vs. Mild PD (H&Y ≤ 2)		
	Controls	PD		Controls	PD
Controls	41	9	Controls	36	14
PD	14	248	PD	11	174

**Table 3:** Classification results of models in distinguishing controls from all Parkinson’s disease (PD) and mild-PD (H&Y ≤ 2) participants. CI: 95% confidence interval.

Controls vs. PD	All PD	Mild PD
Number of PD participants	262	185
Accuracy (CI) [%]	92.6 (88.8, 94.9)	89.4 (84.3, 92.3)
AUC-ROC (CI)	0.88 (0.83, 0.94)	0.83 (0.77, 0.90)
Sensitivity (CI)	0.95 (0.91, 0.97)	0.94 (0.90, 0.97)
Specificity (CI)	0.82 (0.69, 0.91)	0.72 (0.58, 0.83)
F_1_ score (CI)	0.96 (0.93, 0.97)	0.93 (0.90, 0.96)

**Table 4:** Clinical characteristics of correctly classified and misclassified controls and Parkinson’s disease (PD) participants.

		Controls			PD		
		**False Positive**	**True Negative**	p	**False Negative**	**True Positive**	p
All PD							
	UPDRS_PIII	–	–	–	15.8 ± 8.3	22.5 ± 11.8	0.006
	MoCA	–	–	–	28.6 ± 2.5	27.3 ± 3.0	0.075
	CIRS-G	–	–	–	4.7 ± 4.3	4.9 ± 3.4	0.88
	Age	70.9 ± 7.3	62.6 ± 7.3	0.006	65.9 ± 6.8	66.9 ± 9.3	0.30
	Sex (% male)	11.1	36.0	0.10	50.0	63.3	0.33
	Medication state (% ON)	–	–	–	85.7	70.6	0.44
	H&Y (n)			–			0.47
	1				1	11	
	1.5				1	3	
	2				10	159	
	2.5				1	34	
	3				1	24	
	4				0	17	
Mild PD							
	UPDRS_PIII	–	–	–	13.7 ± 7.4	18.7 ± 9.6	0.03
	MoCA	–	–	–	28.1 ± 1.9	27.5 ± 2.8	0.36
	CIRS-G	–	–	–	4.9 ± 4.4	4.4 ± 3.1	0.72
	Age	71.5 ± 6.3	61.3 ± 6.3	4.0 × 10^−5^	65.2 ± 9.1	69.2 ± 7.5	0.12
	Sex (% male)	42.9	36.1	0.71	45.5	60.9	0.32
	Medication state (% ON)	–	–	–	88.2	71.3	0.36
	H&Y (n)			–			0.38
	1				1	11	
	1.5				1	3	
	2				9	160	

**Table 5:** Accuracy (%) of super-learner models using unsegmented tasks and a combination of segmented tasks and calculated kinesiological features to distinguish controls from PD participants. The numbers in parentheses represent the difference in accuracy between each model and the corresponding standard model, which used data from segmented tasks without incorporating kinesiological features (same row).

	With Segmented Tasks and No Kinesiological Features	With Unsegmented Tasks and No Kinesiological Features	With Segmented Tasks and Kinesiological Features
All PD vs. controls	92.6	89.4 (−3.2)	87.8 (−4.8)
Mild PD (H&Y ≤ 2) vs. controls	89.4	84.3 (−5.1)	88.9 (−0.50)

**Table 6:** Accuracy (%) of alternative models. TUG-only and cogTUG-only models used features derived from the first trial, the second trial, and the mean of corresponding features from both trials. TUG-duration and cogTUG-duration models used the durations of TUG and cogTUG tasks. The numbers in parentheses represent the difference in accuracy between the respective model and the corresponding all-task (standard) model (same row).

	All Tasks	TUG-Only	cogTUG-Only	TUG-Duration	cogTUG-Duration
All PD vs. controls	92.6	90.1 (−2.5)	89.4 (−3.2)	83.3 (−9.3)	84.0 (−8.6)
Mild PD (H&Y ≤ 2) vs. controls	89.4	77.9 (−11.7)	87.2 (−2.4)	78.7 (−10.7)	77.0 (−12.4)

## Data Availability

De-identified demographic, clinical, and sensor-derived data have been deposited in the Digital Repository at the University of Maryland (DRUM), https://doi.org/10.13016/dspace/ddzp-im5e. The code used in this research paper is also accessible at the same link. The provided notebook contains the code used to implement each component of the signal processing and machine learning pipelines. Detailed instructions on setting up the necessary environment and running the code are provided in the README file.

## Supplementary Materials

The following supporting information can be downloaded at: https://www.mdpi.com/article/10.3390/s24154983/s1, Figure S1: (a) An example signal for the angular velocity around the roll axis (gx, black line) and the trapezoidal integration of the signal (red line) for the TUG task. Segments (1) and (2) represent the two turns. (b) The signal for the vertical acceleration (az, black line) and the trapezoidal integration of the signal (red line). Segments (1) and (2) represent the sit-to-stand and stand-to-sit parts. Figure S2: Example results of our segmentation approach for the TUG task applied to all six channels. The components are (1) sit-to-stand, (2) first walk, (3) first turn, (4) second walk, (5) second turn, and (6) stand-to-sit. Figure S3: Example results of our segmentation approach for the 32-foot walk task applied to all six channels. The components are (1) first walk, (2) first turn, (3) second walk, (4) second turn, (5) third walk, (6) third turn, and (7) fourth walk. Figure S4: Feature importance scores (left) and SHAP values (right) of the features included in the all PD vs controls classification model. The first column conveys the task and signal channel. Features are ranked by their importance defined as the mean decrease in area under the curve (AUC) between the original and permuted models. SHAP values were calculated using the kernel SHAP method (see Supplementary Methods). Points in the SHAP plot represent participants from the test sets and SHAP values indicate the impact on model output. The color gradient represents the variable values normalized based on percentile ranks. Figure S5: Correlation matrix of the features included in the all PD vs. controls classification mode. Figure S6: Feature importance scores (left) and SHAP values (right) of the features included in the mild PD (H&Y values ≤ 2) vs. controls classification model. The first column conveys the task and signal channel. Features are ranked by their importance defined as the mean decrease in area under the curve (AUC) between the original and permuted models. SHAP values were calculated using the kernel SHAP method (see Supplementary Methods). Points in the SHAP plot represent participants from the test sets and SHAP values indicate the impact on model output. The color gradient represents the variable values normalized based on percentile ranks. Figure S7: Base model coefficients in final super learner classifier using all PD participants and controls. Models with zero coefficients are not shown. Figure S8: Base model coefficients in final super learner classifier using mild PD (H&Y ≤ 2) participants and controls. Models with zero coefficients are not shown. Figure S9: Feature importance scores (left) and SHAP values (right) of the features included in the moderate PD (H&Y = 2.5, 3) vs. controls classification model. The first column conveys the task and signal channel. Features are ranked by their importance defined as the mean decrease in AUC between the original and permuted models. SHAP values were calculated using the kernel SHAP method (see Supplementary Methods). Points in the SHAP plot represent participants from the test sets and SHAP values indicate the impact on model output. The color gradient represents the variable values normalized based on percentile ranks. Figure S10: Feature importance scores (left) and SHAP values (right) of the features included in the severe PD (H&Y = 4) vs. controls classification model. The first column conveys the task and signal channel. Features are ranked by their importance defined as the mean decrease in AUC between the original and permuted models. SHAP values were calculated using the kernel SHAP method (see Supplementary Methods). Points in the SHAP plot represent participants from the test sets and SHAP values indicate the impact on model output. The color gradient represents the variable values normalized based on percentile ranks. Figure S11: Base model coefficients in final super learner classifier using moderate PD (H&Y 2.5 and 3) and controls. Models with zero coefficients are not shown. Figure S12: Base model coefficients in final super learner classifier using severe PD (H&Y 4) and controls. Models with zero coefficients are not shown. Figure S13: Group feature importance of models distinguishing (a) moderate (H&Y = 2.5, 3) Parkinson’s disease (PD) and (b) severe PD (H&Y = 4) participants from controls. Groups are ranked by their importance calculated by permuting features within each group simultaneously and reporting the mean decrease in accuracy between the original and permuted models. Under the null hypothesis that there is no association of the group of predictor variables and the model prediction, permutation should have no or little impact on predictive performance. Figure S14: Comparison of the total duration of (a) TUG and (b) cogTUG tasks between different groups of PD participants and controls. Different PD groups shows significantly longer durations when compared to the controls using two-tailed Student’s *t*-test (*p* < 0.0014). Table S1: Number of sensors and mobility tasks used in a survey of literature from 2017 to 2024. Table S2: Frequency domain features. The power spectrum is obtained using the Discrete Fourier Transform (DFT) of the signal with a fast algorithm, the Fast Fourier Transform (FFT). Table S3: Time domain features. Table S4: Cross-time domain features. Table S5: Features selected by the feature selection approach for TUG-only and cogTUG-only models using features from the first trial, the second trial, and the mean of the corresponding features from both trials. Table S6: Confusion matrix of the classifier using controls and participants with moderate (H&Y 2.5, 3) and severe (H&Y 4) Parkinson’s disease (PD). Rows represent actual class and columns represent predictions. Table S7: Classification results of models distinguishing moderate (H&Y = 2.5, 3) and severe (H&Y = 4) Parkinson’s disease (PD) participants from controls. Table S8: Clinical characteristics of correctly classified and misclassified controls and moderate and severe Parkinson’s disease (PD) participants. Table S9: Accuracy (%) of super learner models using the unsegmented tasks and combination of segmented tasks and calculated kinesiological features to distinguish controls from PD participants. The numbers in parentheses represent the difference in accuracy between each model and the corresponding standard model, which used data from segmented tasks without incorporating kinesiological features (same row). Table S10: Accuracy (%) of alternative models. TUG-only and cogTUG-only models used features derived from the first trial, the second trial, and the mean of corresponding features from both trials. TUG-duration and cogTUG-duration models used the duration of TUG and cogTUG tasks. The numbers in parentheses represent the difference in accuracy between the respective model and the corresponding all-tasks model (same row). Table S11: Comparing the duration of TUG sub-tasks between different groups of PD participants and controls (ctrl). Table S12: Comparing the duration of cogTUG sub-tasks between different groups of PD participants and controls (ctrl). References [170,171,172,173,174,175,176,177,178,179,180,181,182,183,184,185,186,187,188,189,190] are cited in the Supplementary Materials.
