# Supplementary material for: Simplification of Mobility Tests and Data Processing to Increase Applicability of Wearable Sensors as Diagnostic Tools for Parkinson’s Disease
Source: Sensors (Basel). 2024 Aug 1;24(15):4983. doi: 10.3390/s24154983 (PMC11314738; doi:10.3390/s24154983)
Supplement: Supplementary file 1 [file sensors-24-04983-s001.zip › sensors-3062570-supplementary.pdf]

# Supplementary Material for Simplification of Mobility Tests and Data Processing to Increase Applicability of Wearable Sensors as Diagnostic Tool for Parkinson's Disease

**Rana M. Khalil [1], Lisa M. Shulman [2], Ann L. Gruber-Baldini [3], Sunita Shakya [3], Rebecca Fenderson [2,†], Maxwell Van Hoven [2,‡], Jeffrey M. Hausdorff [4,5,6,7,8], Rainer von Coelln [2,*] and Michael P. Cummings [1,*]**

1 Center for Bioinformatics and Computational Biology, University of Maryland, College Park, MD 20742, USA; rmkhalil@umd.edu
2 Department of Neurology, University of Maryland School of Medicine, Baltimore, MD 21201, USA; lshulman@som.umaryland.edu (L.M.S.); rfenders12@gmail.com (R.F.); mvanhoven2@gmail.com (M.V.H.)
3 Department of Epidemiology and Public Health, University of Maryland School of Medicine, Baltimore, MD 21201, USA; abaldin@som.umaryland.edu (A.L.G.-B.); sshakya@som.umaryland.edu (S.S.)
4 Center for the Study of Movement, Cognition, and Mobility, Neurological Institute, Tel Aviv Medical Center, Tel Aviv 6492416, Israel; jeffh@tlvmc.gov.il
5 Department of Physical Therapy, Faculty of Medicine & Health Sciences, Tel Aviv University, Tel Aviv 6997801, Israel
6 Sagol School of Neuroscience, Tel Aviv University, Tel Aviv 6997801, Israel
7 Rush Alzheimer's Disease Center, Rush University Medical Center, Chicago, IL 60612, USA
8 Department of Orthopedic Surgery, Rush University Medical Center, Chicago, IL 60612, USA
* Correspondence: rvoncoelln@som.umaryland.edu (R.v.C.); mcummin1@umd.edu (M.P.C.)
† Current address: Department of Emergency Medicine, Cooper Medical School, Rowan University, Camden, NJ 08103, USA.
‡ Current address: Department of Family Medicine, The Warren Alpert Medical School, Brown University, Pawtucket, RI 02912, USA.

# Contents

**S1 Supplementary Methods** 14
S1.1 SHAP values and individual feature importance . . . 14

**S2 Supplementary Results** 15
S2.1 Classification of moderate and severe PD versus control . . . 15
S2.2 Feature-level importance . . . 20
S2.3 Single feature-based models: total TUG/cogTUG duration . . . 21

**S3 Supplementary Files** 23

# Supplementary Tables

S1 Table S1 . . . 3
S2 Table S2 . . . 4
S3 Table S3 . . . 6
S4 Table S4 . . . 9
S5 Table S5 . . . 10
S6 Table S6 . . . 17
S7 Table S7 . . . 17
S8 Table S8 . . . 19
S9 Table S9 . . . 19
S10 Table S10 . . . 20
S11 Table S11 . . . 22
S12 Table S12 . . . 22

# Supplementary Figures

S1 Figure S1 . . . 11
S2 Figure S2 . . . 11

S3 Figure S3 . . . 12
S4 Figure S4 . . . 12
S5 Figure S5 . . . 13
S6 Figure S6 . . . 13
S7 Figure S7 . . . 14
S8 Figure S8 . . . 14
S9 Figure S9 . . . 15
S10 Figure S10 . . . 16
S11 Figure S11 . . . 16
S12 Figure S12 . . . 17
S13 Figure S13 . . . 18
S14 Figure S14 . . . 21

**Table S1.** Number of sensors and mobility tasks used in a survey of literature from 2017 to 2024.

| Objective | Authors | No. of sensors | No. of mobility tasks |
|---|---|---|---|
| PD diagnosis | Schlachetzki et al. [1] | 2 | 1 |
| | Krupicka et al. [2] | 2 | 3 |
| | Abtahi et al. [3] | 17 | 8 |
| | Castaño-Pino et al. [4] | 18 | 1 |
| | Phan et al. [5] | 4 | 4 |
| | Del et al.Din et al. [6] | 1 | 4 |
| | Rovini et al. [7] | 1 | 4 |
| | Rehman et al. [8] | 2 | 2 |
| | Buckley et al. [9] | 3 | 1 |
| | Cai et al. [10] | 10 | 1 |
| | Jovanovic et al. [11] | 16 | 1 |
| | Battista et al. [12] | 1 | 4 |
| | Yue et al. [13] | 5 | 12 |
| | Bailo et al. [14] | 1 | 1 |
| | Nair et al. [15] | 16 | 1 |
| | Keloth et al. [16] | 2 | 3 |
| | Trabassi et al. [17] | 1 | 1 |
| | Marin et al. [18] | 1 | 5 |
| | Gourrame et al. [19] | 6 | 1 |
| | Meng et al. [20] | 11 | 1 |
| | Caramia et al. [21] | 8 | 1 |
| Quantifying/monitoring the severity of PD symptoms | Mahadevan et al. [22] | 1 | 14 |
| | Lin et al. [23] | 1 | 8 |
| | Borzì et al. [24] | 1 | 1 |
| | Ymeri et al. [25] | 1 | 5 |
| | Bobic et al. [26] | 2 | 1 |
| | Han [27] | 2 | 1 |
| | Heijmans et al. [28] | 6 | 1 |
| | Wu et al. [29] | 6 | 1 |
| | Wu et al. [30] | 6 | 1 |
| | Pugh [31] | 6 | 1 |
| | Sotirakis et al. [32] | 6 | 2 |
| | Delrobaei et al. [33] | 17 | 2 |
| | Memar et al. [34] | 17 | 2 |
| | di Biase et al. [35] | 5 | 3 |
| | Delrobaei et al. [36] | 17 | 3 |
| | Singh et al. [37] | 4 | 5 |
| | Prakash et al. [38] | 4 | 5 |
| | Bremm et al. [39] | 2 | 6 |
| | Huo et al. [40] | 8 | 6 |
| | Yan et al. [41] | 8 | 6 |
| | Hssayeni et al. [42] | 2 | 8 |
| | Zajki-Zechmeister et al. [43] | 2 | 8 |
| | Lonini et al. [44] | 6 | 13 |
| | Tsakanikas et al. [45] | 5 | 5 |
| Detecting freezing of gait in PD | Chomiak et al. [46] | 1 | 4 |
| | Punin et al. [47] | 1 | 3 |
| | Li et al. [48] | 1 | 3 |
| | Camps et al. [49] | 1 | 3 |
| | Samà et al. [50] | 1 | 4 |
| | Capecci et al. [51] | 1 | 3 |
| | Reches et al. [52] | 3 | 1 |
| | Pham et al. [53] | 3 | 1 |
| | Masiala et al. [54] | 3 | 3 |
| | Mancini et al. [55] | 8 | 2 |
| | Marcante et al. [56] | 13 | 5 |
| | Sigcha et al. [57] | 1 | 7 |
| | Bikias et al. [58] | 1 | 3 |
| | Pardoel et al. [59] | 4 | 2 |
| | Shi et al. [60] | 3 | 2 |
| Quantitative characterization of gait/ identifying imbalance in PD | Rennie et al. [61] | 1 | 3 |
| | Myers et al. [62] | 1 | 3 |
| | Zadka et al. [63] | 1 | 3 |
| | Haji et al.Ghassemi et al. [64] | 2 | 1 |
| | Nguyen et al. [65] | 2 | 1 |
| | Castelli Gattinara Di Zubiena et al. [66] | 3 | 1 |
| | Liuzzi et al. [67] | 3 | 1 |
| | Stack et al. [68] | 5 | 7 |
| | Jehu et al. [69] | 7 | 2 |
| | Zhang et al. [70] | 8 | 2 |
| | Romijnders et al. [71] | 2 | 3 |
| | Lukšys et al. [72] | 6 | 1 |

**Table S2.** Frequency domain features. The power spectrum is obtained using the Discrete Fourier Transform (DFT) of the signal with a fast algorithm, the Fast Fourier Transform (FFT) [73]. Variables: $f_j$ is the frequency of the spectrum at frequency bin $j$; $P_j$ is the power spectrum at frequency bin $j$; and $M$ is the length of the frequency bin.

| Name | Description | Mathematical equation | References |
|---|---|---|---|
| MNF | Mean frequency | $\sum_{j=1}^{M} f_j P_j \;/\; \sum_{j=1}^{M} P_j$ | [74, 75, 76] |
| MDF | Median frequency | $\frac{1}{2}\sum_{j=1}^{M} P_j$ | [74, 75, 76] |
| PSD | Power spectrum deformation | $\frac{\sqrt{\frac{M_2}{M_0}}}{\frac{M_1}{M_0}}$ ; $M_n = \sum_{j=1}^{M} f_j P_j$ | [75, 77] |
| FI | Freeze index | $\sum_{j=3}^{8} P_j \;/\; \sum_{j=0.5}^{3} P_j$ | [78, 76] |
| ENT | Entropy | $-\sum_{j=1}^{M} Pr_j log(Pr_j)$ ; $Pr_j = \frac{P_j}{\sum_{j=1}^{M} P_j}$ | – |
| TTP | Total power | $\sum_{j=1}^{M} P_j$ | [74, 76] |
| MNP | Mean power | $\sum_{j=1}^{M} P_j \;/\; M$ | [74, 76] |
| PKF | Peak frequency | $f_{argmax(P_j)\,,\, j=1...M}$ | [74, 76] |
| PKF | Peak frequency | $max(P_j)$ , $j = 1...M$ | [79] |
| FR | Frequency ratio | $\frac{max(f_j)}{min(f_j)}$ , $j = 1...M$ | [74, 76] |
| PSR | Power spectrum ratio | $\frac{P_0}{P} = \sum_{f_0-n}^{f_0+n} P_j \;/\; \sum_{j=-\infty}^{\infty} P_j$ <br> $f_0$: value of $PKF$, $n$: integral limit. | [74] |
| SM | Spectral moments | $SM_i = \sum_{j=1}^{M} f_j^i P_j$ , $i = 1, 2, 3$ | [74, 77, 76] |
| VR | Variance | $\frac{1}{M-1}\sum_{j=1}^{M}(P_j - MNP)^2$ | – |
| SD | Standard deviation | $\sqrt{\frac{1}{M-1}\sum_{j=1}^{M}(P_j - MNP)^2}$ | – |
| SS | Skewness | $\frac{1}{M-1}\sum_{j=1}^{M}(P_j - MNP)^3 \;/\; SD^3$ | – |
| SK | Kurtosis | $\frac{1}{M-1}\sum_{j=1}^{M}(P_j - MNP)^4 \;/\; SD^4$ | – |
| SBW | Spectral bandwidth | $\frac{\sum_{j=1}^{M}(f_j - MNF)^2 P_j}{TTP}$ | – |
| SR | Spectral roll-off | $c\sum_{j=1}^{M} P_j$ | – |
| VCF | Variance of central frequency | $\frac{SM_2}{SM_0} - \left(\frac{SM_1}{SM_0}\right)^2$ | [74] |

| | | | |
|---|---|---|---|
| MSE | Mean spectral energy | $\sum_{j=1}^{M} \|P_j\|^2 \ / \ M$ | [80, 81] |
| DPR | Maximum to minimum drop in power density | $\frac{\text{highest mean power density value}}{\text{lowest mean power density value}}$ | [77, 75] |
| SN | Signal to noise ratio | $\frac{\sum P_j \text{ in the upper 20\% frequency range}}{TTP}$ | [77, 75] |
| HR | Harmonic ratio | $\frac{\sum \text{amplitudes of even harmonics}}{\sum \text{amplitudes of odd harmonics}}$ | [9] |

**Table S3.** Time domain features. Variables: $x_n$ represents $n^{th}$ sample of the signal; and $N$ is the length of the signal.

| Name | Description | Mathematical equation | References |
|---|---|---|---|
| MN | Mean | $\frac{1}{N}\sum_{n=1}^{N} x_n$ | [82, 75, 83] |
| VR | Variance | $\frac{1}{N-1}\sum_{n=1}^{N}(x_n - MN)^2$ | [75] |
| SD | Standard deviation | $\sqrt{\frac{1}{N-1}\sum_{n=1}^{N}(x_n - MN)^2}$ | [82, 75, 83] |
| SS | Skewness | $\frac{1}{N-1}\sum_{n=1}^{N}(x_n - MN)^3 \,/\, SD^3$ | [82, 75, 83] |
| SK | Kurtosis | $\frac{1}{N-1}\sum_{n=1}^{N}(x_n - MN)^4 \,/\, SD^4$ | [82, 75, 83] |
| IAV | Integrated absolute value | $\sum_{n=1}^{N} \lvert x_n \rvert$ | [74, 76] |
| MAV | Mean absolute value | $\frac{1}{N}\sum_{n=1}^{N} \lvert x_n \rvert$ | [74, 76] |
| SSI | Simple square interval | $\sum_{n=1}^{N} \lvert x_n \rvert^2$ | [74, 76] |
| RMS | Root mean square | $\sqrt{\frac{1}{N}\sum_{n=1}^{N} x_n^2}$ | [74, 76] |
| V3 | V-order 3 | $\sqrt[3]{\frac{1}{N}\sum_{n=1}^{N} \lvert x_n \rvert^3}$ | [74, 76] |
| WL | Waveform length | $\sum_{n=1}^{N-1} \lvert x_{n+1} - x_n \rvert$ | [74, 75, 76] |
| AAC | Average amplitude change | $\frac{1}{N}\sum_{n=1}^{N-1} \lvert x_{n+1} - x_n \rvert$ | [74, 76] |
| DASDV | Difference absolute standard deviation value | $\sqrt{\frac{1}{N-1}\sum_{n=1}^{N-1}(x_{n+1} - x_n)^2}$ | [74, 76] |
| MFL | Maximum fractal length | $log_{10}\left(\sqrt{\sum_{n=1}^{N-1}(x_{n+1} - x_n)^2}\right)$ | [76, 84] |

| | | | |
|---|---|---|---|
| ZC | Zero crossing | $\sum_{n=1}^{N-1} sgn(x_n * x_{n+1}) \cap \|x_n - x_{n+1}\| \geq threshold$ <br> $sgn(x) = 1 \quad if\ x \geq threshold;\ \ 0\ \ o.w$ | [74, 75] |
| RC | Rate of change | $\sum_{n=1}^{N-1} f\|x_n - x_{n+1}\|$ <br> $f(x) = 1 \quad if\ x \geq threshold;\ \ 0\ \ o.w$ | [74, 75] |
| SSC | Slope sign change | $\sum_{n=2}^{N-1} f[(x_n - x_{n-1}) * (x_n - x_{n+1})]$ <br> $f(x) = 1 \quad if\ x \geq threshold;\ \ 0\ \ o.w$ | [74, 75] |
| DR | Data range | $max(x_n) - min(x_n)\ ;\ n = 1...N$ | [82] |
| ENT | Entropy | $-\sum_{b=1}^{numBins} Pr_b log(Pr_b)\ ;\ numBins = 10$ | [82] |
| LOG | Log detector | $e^{\frac{1}{N}\sum_{n=1}^{N} log\|x_n\|}$ | [74] |
| MAD | Mean absolute deviation | $\frac{1}{N}\sum_{n=1}^{N} \|x_n - MN\|$ | [75] |
| Q1, Q2, Q3 | 1st, 2nd, 3rd quartiles | | [82] |
| IQR | Interquartile range | $Q_3 - Q_1$ | [82, 83] |
| CV | Coefficient of variation | $SD/MN$ | [85, 86] |
| MD | Median | | [82] |
| MOD | Mode | | [82] |
| TKEO | Teager-Kaiser energy operator | $mean(x_n^2 - (x_{n-1} * x_{n+1}))\ ;\ n = 1...N$ | [87, 82] |
| AR | Auto-regressive coefficients | $x_i = \sum_{p=1}^{P} a_p x_{i-p} + w_i$ <br> $P = 4$ , $a_i$ : AR coefficient , $w_i$ : noise term | [74, 75] |

| | | | |
|---|---|---|---|
| DFA | Detrended fluctuation analysis | $y_k = \sum_{t=1}^{k} x_n - MN$ ; $k = 1...N$<br>$F_{s,t} = \sqrt{\frac{1}{N}\sum_{k=1}^{N}(y_k - y_{k,s})^2}$ ;<br>$s = 1...N_t$ , $N_t = N/t$ ,<br>$t$ : num of nonoverlapping segments ,<br>$y_{k,s}$ : fitting polynomial in segment $s$<br>$DFA = F(t) = \left[\frac{1}{2N_t}\sum_{s=1}^{2N_t} F_{s,t}^2\right]^{1/2}$ | [88, 82, 84] |
| HFD | Higuchi's fractal dimension | $X_k^n = X(n+ik)$ ; $i \in [0, [(N-n)/k]]$ ,<br>$n \in [1,k]$ , $n$ : initial time , $k$ : time interval<br>$L_n(k) = \frac{\left(\sum_{i=1}^{\left[\frac{N-n}{k}\right]} \left\lvert X(n+ik) - X(n+(i-1)k)\right\rvert\right)\frac{N-1}{\left[\frac{N-n}{k}\right]k}}{k}$<br>$L_n(k)$ : length of the curve $X_k^n$<br>$\langle L(k)\rangle \propto k^{-D}$<br>$\langle L(k)\rangle$: length of the curve for time interval k<br>$= mean(L_n(k))$ over $k$ sets ,<br>$D$: fractal dimension , [ ]: Gauss' notation | [84, 89] |
| KATZ | Katz's fractal dimension | $\frac{log(L/a)}{log(d/a)}$<br>$L$ : $\sum$(distances b/w successive points)<br>$a$ : $avg$(distances b/w successive points)<br>$d$ : $max$(distances b/w first and other points) | [90] |
| MAV1 | Modified mean absolute value type 1 | $\frac{1}{N}\sum_{n=1}^{N} w_i \lvert x_n \rvert$<br>$w_i = 1$ $if$ $0.25N \le i \le 0.75N$; $0.5$ $o.w$ | [74] |
| MAV2 | Modified mean absolute value type 2 | $\frac{1}{N}\sum_{n=1}^{N} w_i \lvert x_n \rvert$<br>$w_i = 1$ $if$ $0.25N \le i \le 0.75N$;<br>$4i/N$ $if$ $i < 0.25N$; $4(i-N)/N$ $o.w$ | [74] |
| MAVS | Mean absolute value slope | $MAV_{k+1} - MAV_k$ ; $k = 1...K-1$ , $K = 2$ | [74] |
| MBV | Mean binarized values | $\frac{1}{N}\sum_{n=1}^{N} f(x)$<br>$f(x) = 1$ $if$ $x \ge threshold$; $0$ $o.w$ | [74] |
| TM4 | Absolute temporal moment | $\frac{1}{N}\sum_{n=1}^{N} x^4$ | [74] |

| | | | |
|---|---|---|---|
| VFD | Variation fractal dimension | $\widehat{V}_p(l/N) = \frac{1}{2(N-l)} \sum_{i=l}^{N} \lvert X_{i/N} - X_{(i-l)/N} \rvert^p$ , <br> $\widehat{V}_p$ = moment estimator of order $p$ ; $l = 1, 2, ..$ <br> $\widehat{D}_{V;p} = 2 - \frac{1}{p} \frac{log(\widehat{V}_p(2/N)) - log(\widehat{V}_p(1/N))}{log2}$ <br> $\widehat{D}_{V;p}$: estimator for the fractal dimension | [91] |
| MAX | Maximum | $max(x_n)$ ; $n = 1..N$ | [92] |
| MIN | Minimum | $min(x_n)$ ; $n = 1..N$ | [92] |
| GMN | Geometric mean | $\left(\prod_{n=1}^{N} x_n\right)^{1/N}$ | – |
| HMN | Harmonic mean | $N / \sum_{n=1}^{N} (1/x_n)$ | [93, 94] |
| MDAD | Median absolute deviation | $median(\lvert x_n - MD \rvert)$ | [92] |
| MSENT | Multiscale entropy, scale factor = 4, 5 | $MSE(x, \tau, m, r) = SampEn(y_1^{(\tau)}, m, r)$ <br> $y_{k,j}^{(\tau)} = \frac{1}{\tau} \sum_{i=(j-1)\tau+k}^{j\tau+k-1} x_i$ , $1 \leq j \leq \frac{N}{\tau}$ , $1 \leq k \leq \tau$ <br> $SampEn(x, m, r) = -\ln \frac{n^{m+1}}{n^m}$ <br> $\tau$: scale factor <br> $m$: embedding dimension, $r$: tolerance <br> $n^{m+1}$: # of $(m+1)$ dimensional vectors within $r$ <br> $n^m$: # of $m$ dimensional vectors within $r$ | [95] |

**Table S4.** Cross-time domain features.

| Name | Description | Mathematical equation | References |
|---|---|---|---|
| ENT | Cross-entropy | $H(f, g) = -\int_t f(t)\ \ log(g(t))\ dt$ | – |
| CCP | Cross-correlation | $(f \star g)(t) = \int_{-\infty}^{\infty} \overline{f}(\tau)\ \ g(t+\tau)\ d\tau$ <br> $\overline{f}(\tau)$ is the complex conjugate of $f(\tau)$ | [96] |
| MI | Mutual information | $MI(f; g) = \int_t \int_t P_{(f,g)} log\left(\frac{P_{(f,g)}}{P_f P_g}\right) dt\ dt$ <br> $P_{(f,g)}$ : joint probability mass function of $f$ and $g$ <br> $P_f, P_g$ : marginal probability mass functions of $f$ and $g$ | [97] |

**Table S5.** Features selected by the feature selection approach for TUG-only and cogTUG-only models using features from the first trial, the second trial, and the mean of the corresponding features from both trials.

| | TUG-only | cogTUG-only |
|---|---|---|
| PD vs controls | kurtosis of DFT of sit-to-stand (TUG1, ay), detrended fluctuation analysis of sit-to-stand (TUG1, gz), mean binarized values of stand-to-sit (TUG1, ax), maximum to minimum drop in power density of second turn (TUG1, gy), mean absolute value slope of first turn (TUG2, ax), minimum of second turn (TUG2, gx), frequency at 3rd peak of LSP of stand-to-sit (mean TUG, gy), median frequency of DFT of first walk (mean TUG, az), freeze index of DFT of second walk (mean TUG, az), standard deviation of DFT of second walk (mean TUG, gz), OARS total IADLs score | mean absolute value slope of stand-to-sit (cogTUG2, gx), variation fractal dimension of first turn (cogTUG2, ax), modified mean absolute value type 2 of second turn (cogTUG2, ay), frequency at fifth peak of LSP of sit-to-stand (mean cogTUG, az), frequency at first peak of DFT of stand-to-sit (mean cogTUG, az), peak frequency of DFT of stand-to-sit(mean cogTUG, az), maximum to minimum drop in power density of DFT of stand-to-sit(mean cogTUG, gx), slope sign change of stand-to-sit(mean cogTUG, gz), variation fractal dimension of first turn(mean cogTUG, ax), detrended fluctuation analysis of DFT of first turn(mean cogTUG, az) |
| Mild PD vs controls | spectral bandwidth of DFT of stand-to-sit (TUG1, gx), signal to noise ratio of DFT stand-to-sit (TUG1, gz), frequency of 3rd peak of LSP of stand-to-sit (TUG2, gx), mode of second turn (TUG2, gx), minimum of second turn (TUG2, gx), frequency of 6th peak of LSP of stand-to-sit (mean TUG, gx), frequency of 7th peak of LSP of second turn (mean TUG, gx) | variation fractal dimension of first turn (cogTUG2, ax), root mean square of second turn (cogTUG2, ay), modified mean absolute value type 2 of second turn (cogTUG2, ay), mode of first walk (cogtUG2, ay), slope sign change of stand-to-sit (mean cogTUG, ay), peak frequency of DFT of stand-to-sit (mean cogTUG, az), slope sign change of stand-to-sit (mean cogTUG, az), slope sign change of stand-to-sit (mean cogTUG, gx), slope sign change of stand-to-sit (mean cogTUG, gz) |
| Moderate PD vs controls | waveform length of second walk (TUG1, gx), integrated absolute value of second walk (TUG2, gy), integrated absolute value of second walk (mean TUG, gy), OARS total ADLs score, OARS total IADLs score, OARS total score | geometric mean of first walk (cogTUG1, gx), frequency at 5th peak of LSP of sit-to-stand (cogTUG2, az), mean power of DFT of stand-to-sit (cogTUG2, gy), integrated absolute value of second walk (cogTUG2, gy), simple square interval of second walk (cogTUG2, gy), slope sign change of stand-to-sit (mean cogTUG, az), OARS total ADLs score, OARS total score |
| Severe PD vs controls | slope sign change of first walk (TUG1, ay), frequency at eight peak of LSP of first walk (TUG1, gy), OARS total ADLs score, OARS total IADLs score, OARS total score | integrated absolute value of first walk (cogTUG2, gx), OARS total score |

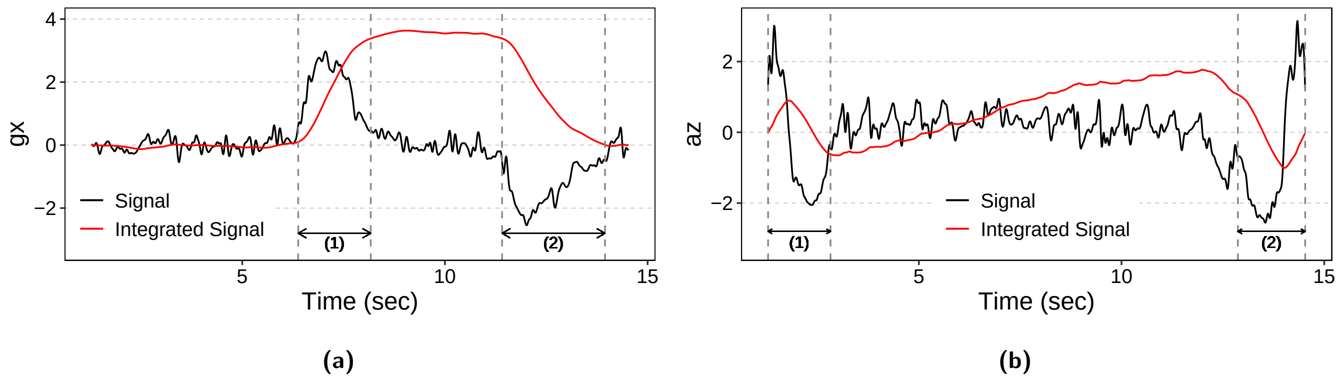


**(a)** **(b)**

**Figure S1.** **(a)** An example signal for the angular velocity around the roll axis (gx, black line) and the trapezoidal integration of the signal (red line) for the TUG task. Segments (1) and (2) represent the two turns. **(b)** The signal for the vertical acceleration (az, black line) and the trapezoidal integration of the signal (red line). Segments (1) and (2) represent the sit-to-stand and stand-to-sit parts.

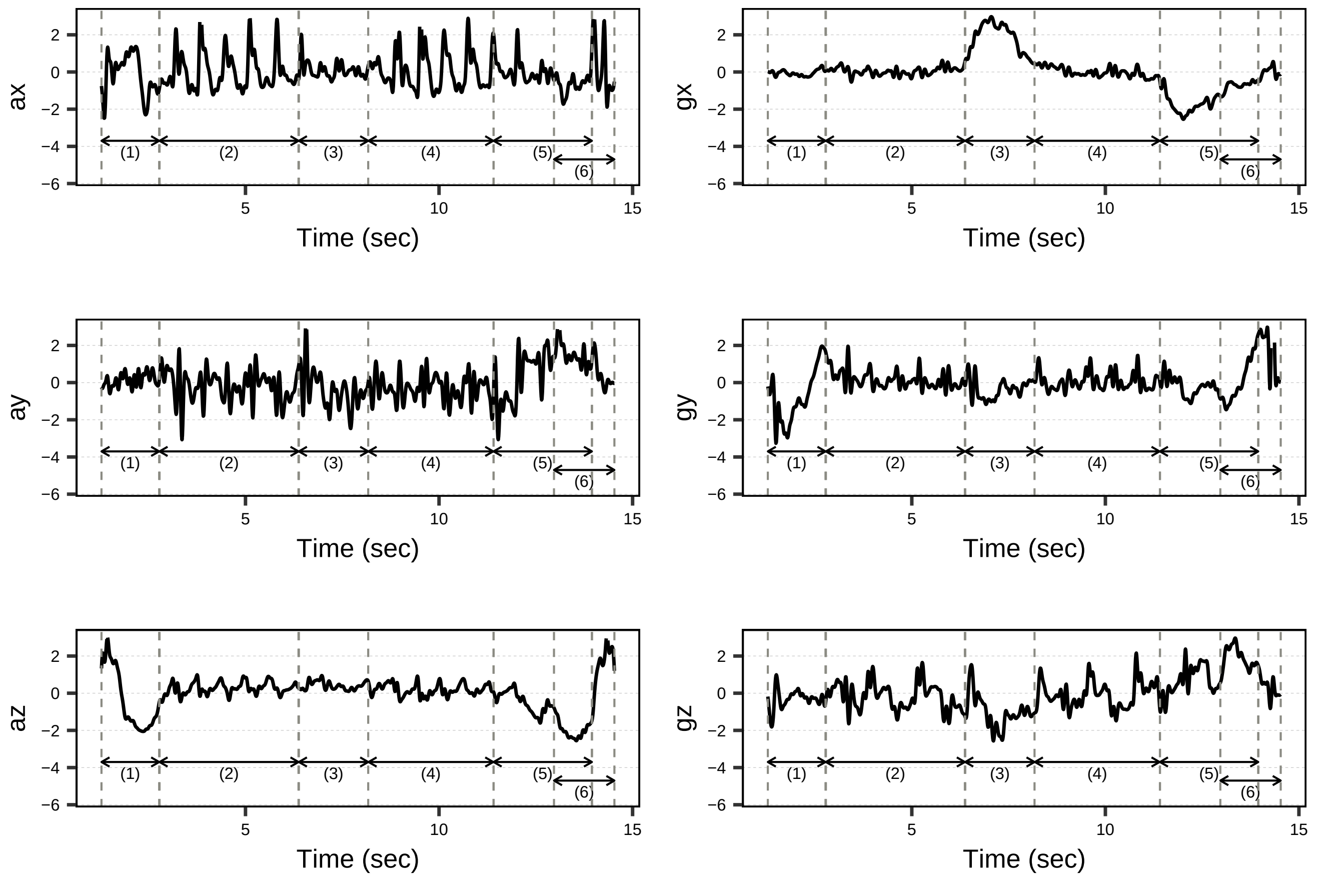


**Figure S2.** Example results of our segmentation approach for the TUG task applied to all six channels. The components are (1) sit-to-stand, (2) first walk, (3) first turn, (4) second walk, (5) second turn, and (6) stand-to-sit.

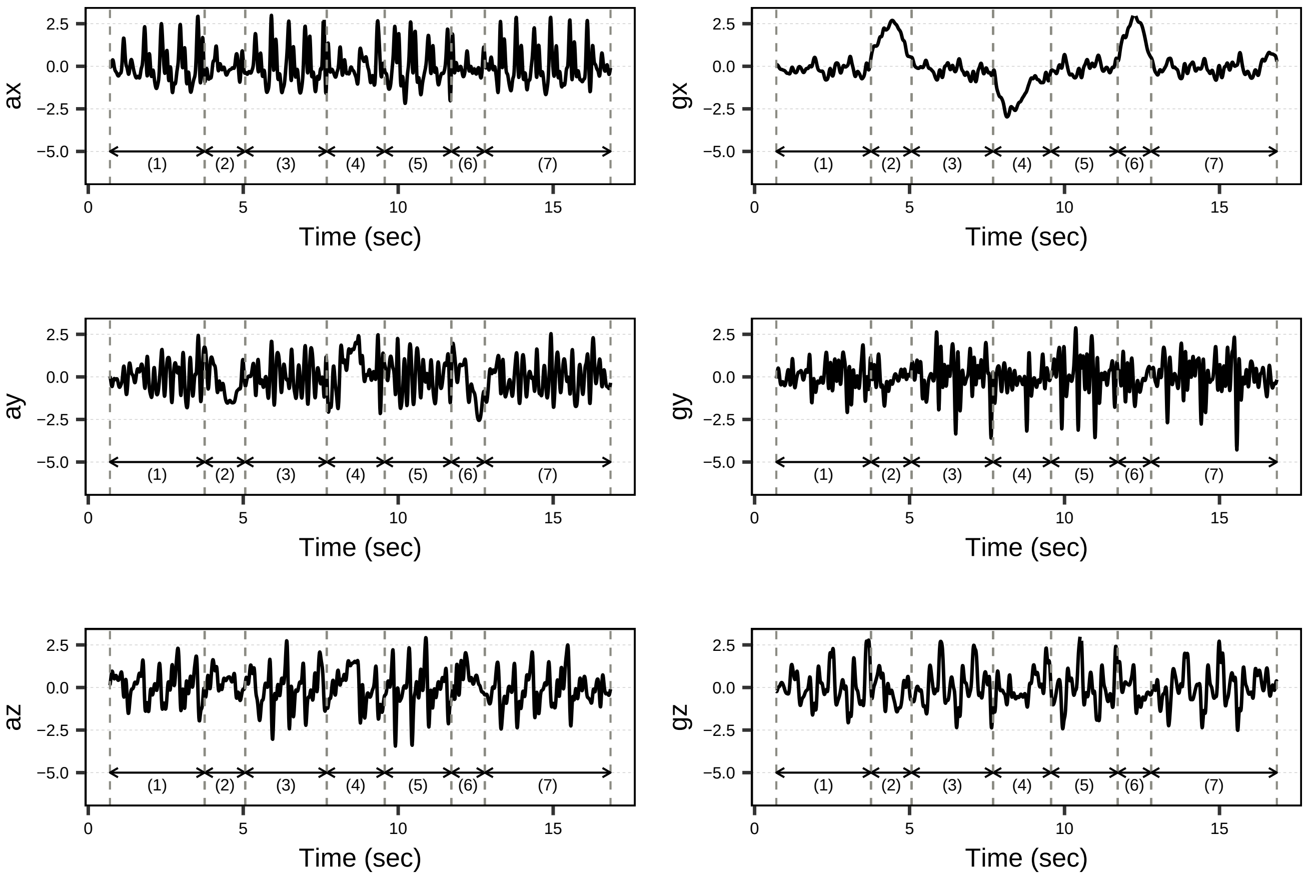


**Figure S3.** Example results of our segmentation approach for the 32-foot walk task applied to all six channels. The components are (1) first walk, (2) first turn, (3) second walk, (4) second turn, (5) third walk, (6) third turn, and (7) fourth walk.

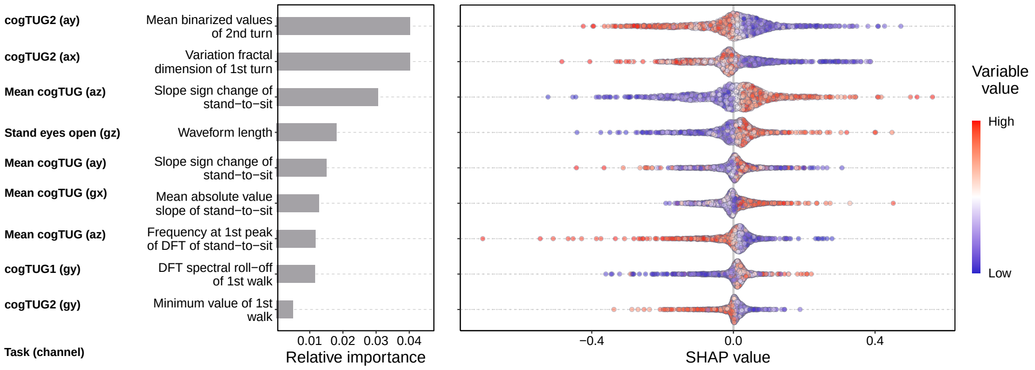


**Figure S4.** Feature importance scores (left) and SHAP values (right) of the features included in the all PD vs controls classification model. The first column conveys the task and signal channel. Features are ranked by their importance defined as the mean decrease in area under the curve (AUC) between the original and permuted models. SHAP values were calculated using the kernel SHAP method (see Supplementary Methods). Points in the SHAP plot represent participants from the test sets and SHAP values indicate the impact on model output. The color gradient represents the variable values normalized based on percentile ranks. DFT: Discrete Fourier Transform.

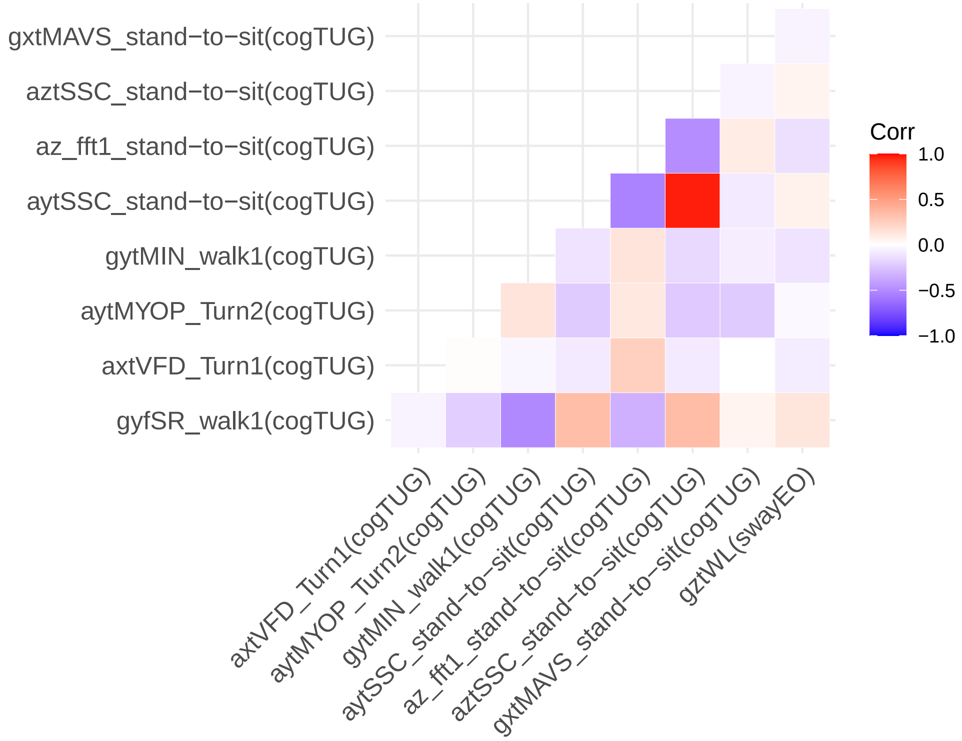


**Figure S5.** Correlation matrix of the features included in the all PD vs controls classification model.

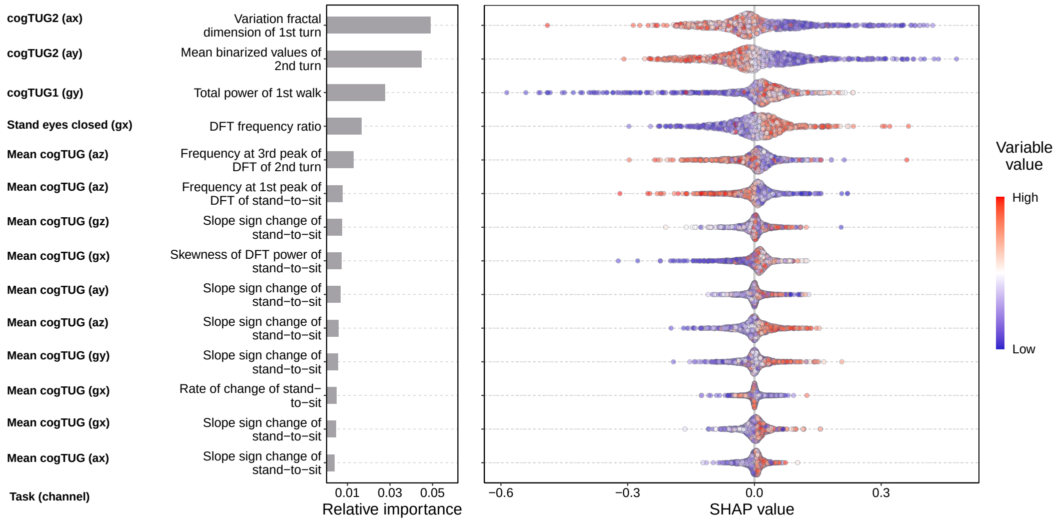


**Figure S6.** Feature importance scores (left) and SHAP values (right) of the features included in the mild PD (H&Y values $\leq 2$) vs controls classification model. The first column conveys the task and signal channel. Features are ranked by their importance defined as the mean decrease in area under the curve (AUC) between the original and permuted models. SHAP values were calculated using the kernel SHAP method (see Supplementary Methods). Points in the SHAP plot represent participants from the test sets and SHAP values indicate the impact on model output. The color gradient represents the variable values normalized based on percentile ranks. DFT: Discrete Fourier Transform.

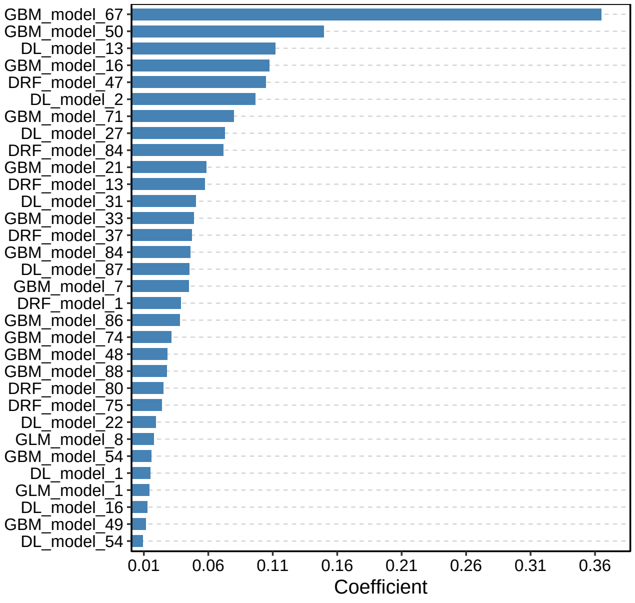


**Figure S7.** Base model coefficients in final super learner classifier using all PD participants and controls. Models with zero coefficients are not shown. GBM: gradient boosting machines, DL: deep learning, DRF: distributed random forests, GLM: generalized linear model.

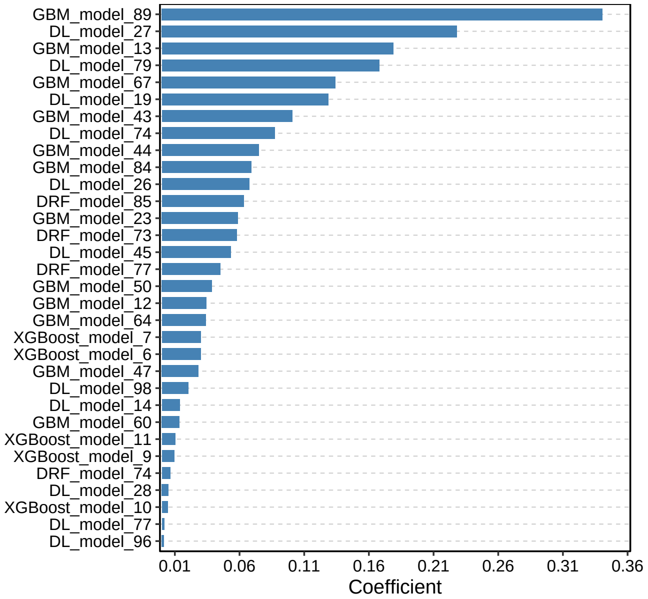


**Figure S8.** Base model coefficients in final super learner classifier using mild PD (H&Y $\leq 2$) participants and controls. Models with zero coefficients are not shown. GBM: gradient boosting machines, DL: deep learning, DRF: distributed random forests, XGBoost: extreme gradient boosting.

# S1 Supplementary Methods

## S1.1 SHAP values and individual feature importance

SHAP (SHapley Additive exPlanations) is a comprehensive approach that applies concepts from game theory to machine learning models for interpretation of results [98]. By assigning a value to each input feature, SHAP illustrates how each feature contributed to the final prediction result. Unlike traditional feature importance metrics,

SHAP considers the impact of features on individual samples, revealing both positive and negative associations with the target variable. To calculate feature importance scores and SHAP values of our classification models, we used the model with the highest contribution weight to the final super learners. By applying the same nested loop framework applied to split the data into training and testing sets, we calculated feature importance scores and SHAP values using the corresponding top model, training, and testing sets. We used the `KernelExplainer` function in the Python package `SHAP` to calculate SHAP values. Kernel SHAP is a model-agnostic method that uses a special weighted linear regression to compute the importance of each feature. Feature importance was defined using a permutation-based scoring scheme where each feature was permuted ten times and the mean decrease in area under the curve (AUC) between the permuted and original models represented the importance score.

# S2 Supplementary Results

## S2.1 Classification of moderate and severe PD versus control

Employing the forward feature selection approach (see Section 2.8), the number of features was reduced from 25,705 to 8 and 4 features for moderate and severe PD classification, respectively. Among the 8 selected features for the moderate classification, 4 were derived from the cogTUG task, one from the TUG task, and 3 were non-sensor features (Supplementary Figure S9). Similarly, for the severe classification, 3 out the 4 selected features were derived from the cogTUG task, and one was a non-sensor feature (Supplementary Figure S10). The selected cogTUG features for both classifiers were derived from the walk, sit-to-stand, and stand-to-sit segments of the task. These features represented acceleration and rotational measures along the vertical, mediolateral, or anteroposterior axes. An extreme gradient boosting machine had the highest contribution to the moderate PD ensemble (Supplementary Figure S11) and a gradient boosting machine had the highest weighted contribution to the final ensemble of severe PD (Supplementary Figure S12).

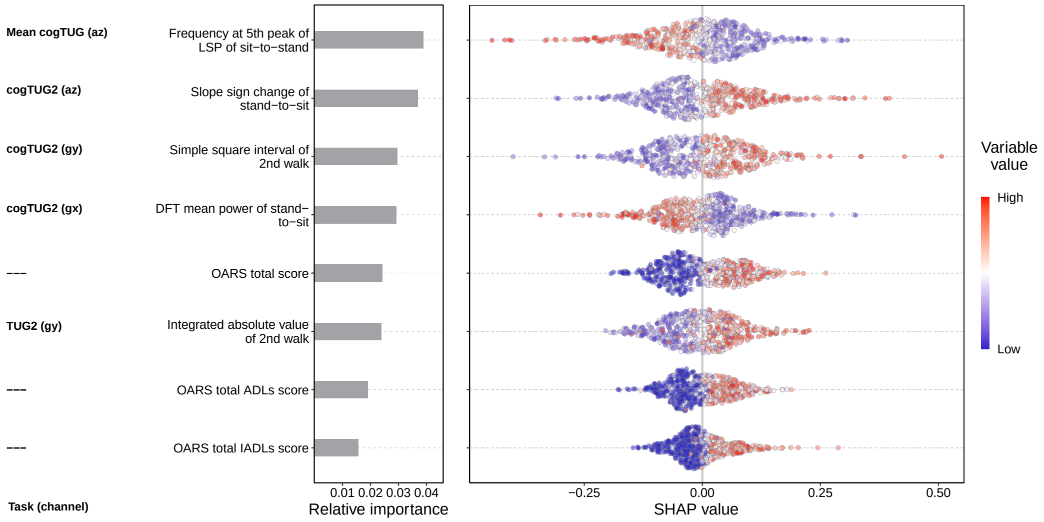


**Figure S9.** Feature importance scores (left) and SHAP values (right) of the features included in the moderate PD (H&Y = 2.5, 3) vs controls classification model. The first column conveys the task and signal channel. Features are ranked by their importance defined as the mean decrease in AUC between the original and permuted models. SHAP values were calculated using the kernel SHAP method (see Supplementary Methods). Points in the SHAP plot represent participants from the test sets and SHAP values indicate the impact on model output. The color gradient represents the variable values normalized based on percentile ranks. DFT: Discrete Fourier Transform, LSP: Lomb-Scargle Periodogram, OARS: Older Americans Resource Survey-Instrumental Activities of Daily Living, ADL: activities of daily living, IADL: instrumental activities of daily living.

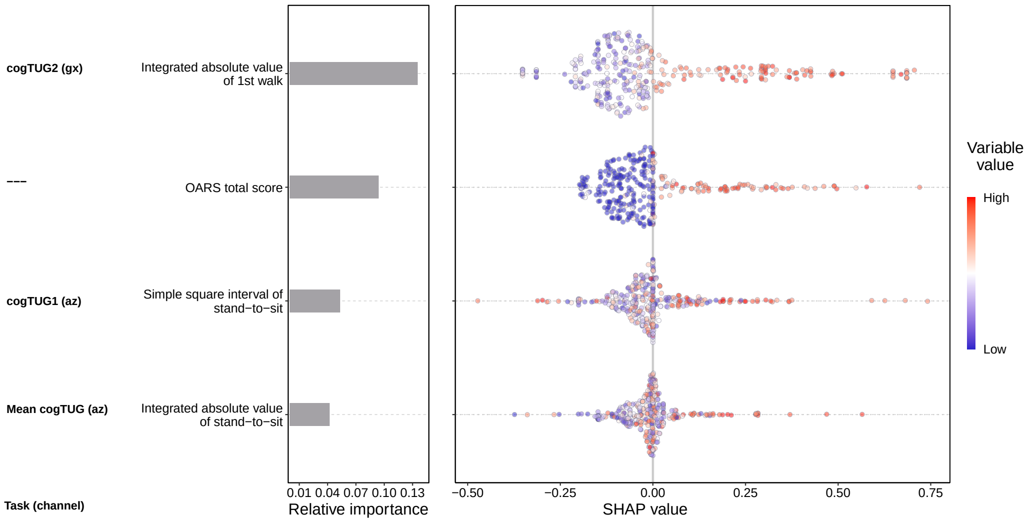


**Figure S10.** Feature importance scores (left) and SHAP values (right) of the features included in the severe PD (H&Y = 4) vs controls classification model. The first column conveys the task and signal channel. Features are ranked by their importance defined as the mean decrease in AUC between the original and permuted models. SHAP values were calculated using the kernel SHAP method (see Supplementary Methods). Points in the SHAP plot represent participants from the test sets and SHAP values indicate the impact on model output. The color gradient represents the variable values normalized based on percentile ranks. OARS: Older Americans Resource Survey-Instrumental Activities of Daily Living.

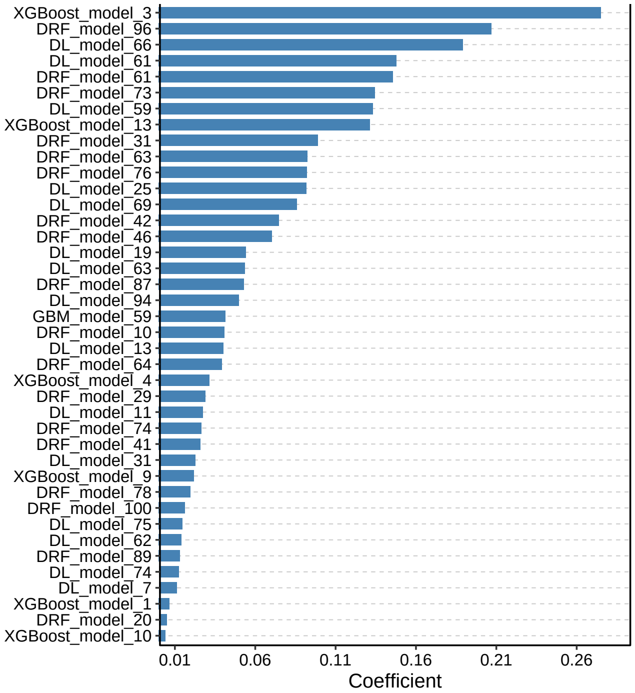


**Figure S11.** Base model coefficients in final super learner classifier using moderate PD (H&Y 2.5 and 3) and controls. Models with zero coefficients are not shown. DL: deep learning, DRF: distributed random forests, XGBoost: extreme gradient boosting.

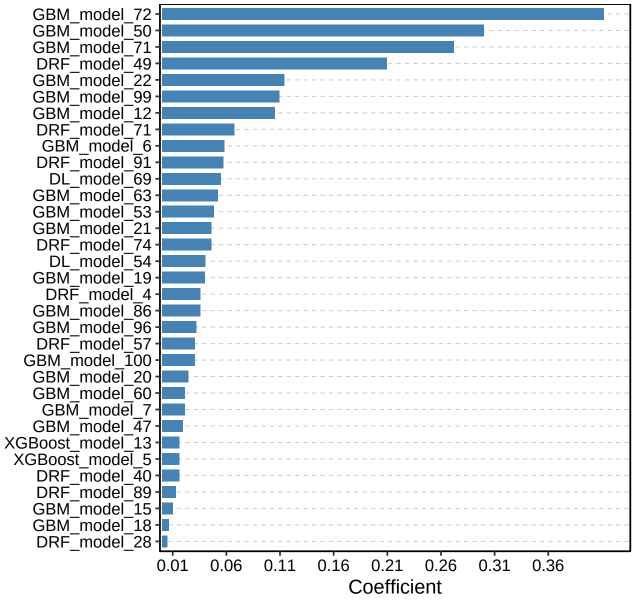


**Figure S12.** Base model coefficients in final super learner classifier using severe PD (H&Y 4) and controls. Models with zero coefficients are not shown. GBM: gradient boosting machines, DL: deep learning, DRF: distributed random forests, XGBoost: extreme gradient boosting.

The accuracy of the classifiers was 90.0% (confidence interval [CI] 81.8%, 93.6%) in distinguishing moderate PD from controls and 97.0% (CI 86.6%, 98.5%) in distinguishing those with severe PD from controls. Supplementary Tables S6 and S7 display confusion matrices and performance metrics for the classifiers.

**Table S6.** Confusion matrix of the classifier using controls and participants with moderate (H&Y 2.5, 3) and severe (H&Y 4) Parkinson's disease (PD). Rows represent actual class and columns represent predictions.

**(a)** Controls vs Moderate PD

| | Controls | PD |
|---|---|---|
| Controls | 44 | 6 |
| PD | 5 | 55 |

**(b)** Controls vs Severe PD

| | Controls | PD |
|---|---|---|
| Controls | 50 | 0 |
| PD | 2 | 15 |

**Table S7.** Classification results of models distinguishing moderate (H&Y = 2.5, 3) and severe (H&Y = 4) Parkinson's disease (PD) participants from controls. CI: 95% confidence interval.

| PD vs controls | Moderate PD | Severe PD |
|---|---|---|
| Number of PD | 60 | 17 |
| Accuracy (CI) [%] | 90.0 (81.8, 93.6) | 97.0 (86.6, 98.5) |
| AUC-ROC (CI) | 0.90 (0.84, 0.96) | 0.94 (0.86, 1.0) |
| Sensitivity (CI) | 0.92 (0.82, 0.97) | 0.88 (0.63, 1.0) |
| Specificity (CI) | 0.88 (0.76, 0.95) | 1.0 |
| $F_1$ score (CI) | 0.91 (0.84, 0.95) | 0.94 (0.75, 1.0) |

Analogous to the findings presented earlier for the all PD and mild PD classifiers, an analysis of groupwise feature importance across the five mobility tasks and non-sensor features revealed that features derived from the cogTUG task were by far the most important in distinguishing individuals with PD at different severity levels from control participants. There was a substantial decline of at least 38% in importance when comparing the cogTUG task to other mobility tasks (Supplementary Figure S13). Additionally, we conducted a detailed analysis of indi-

vidual feature-level importance scores and SHAP values for the moderate and severe PD classifiers (Supplementary Figures S9 and S10, Supplementary Results (Section S2.2)).

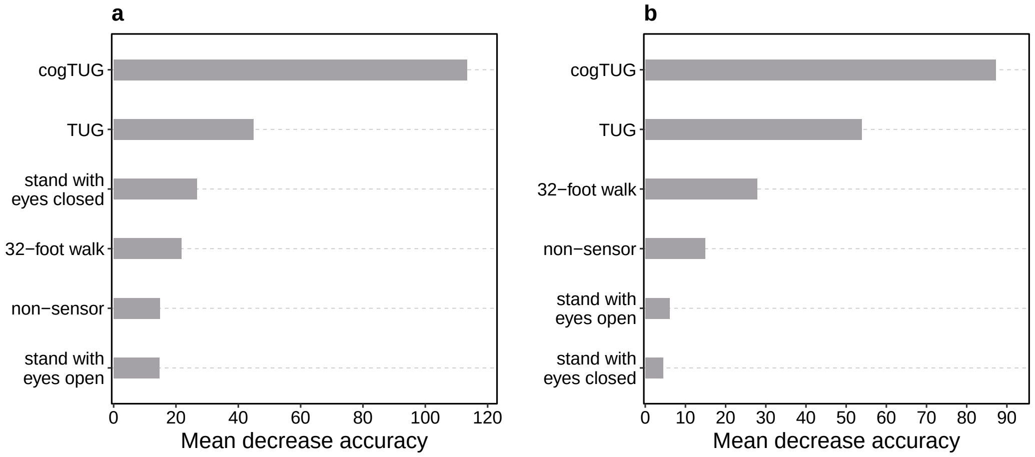


**Figure S13.** Group feature importance of models distinguishing **(a)** moderate (H&Y = 2.5, 3) Parkinson's disease (PD) and **(b)** severe PD (H&Y = 4) participants from controls. Groups are ranked by their importance calculated by permuting features within each group simultaneously and reporting the mean decrease in accuracy between the original and permuted models. Under the null hypothesis that there is no association of the group of predictor variables and the model prediction, permutation should have no or little impact on predictive performance. More detailed individual feature-level importance scores and SHapley Additive exPlanations (SHAP) values are illustrated in Supplementary Figures S9 and S10.

Comparing demographic and clinical characteristics between correctly classified and misclassified participants (see Supplementary Table S8), we found that there was no significant age difference between the correctly classified controls and those falsely classified as moderate PD. No control participants were falsely classified as severe PD. Likewise, there was no significant age difference between correctly classified PD participants (TP) and those falsely classified as controls (FN), regardless of PD severity. UPDRS_PIII motor scores of PD participants falsely classified as controls were lower than in correctly classified participants at moderate and severe H&Y stages, although these differences were not statistically significant. In contrast, MoCA scores were higher for the PD participants falsely classified as controls compared to the correctly classified participants with significant differences for severe PD. None of the other demographic and clinical variables (H&Y stage, medication state, sex, CIRS-G) were significantly different between the correctly and falsely classified PD participants, regardless of PD severity.

**Table S8.** Clinical characteristics of correctly classified and misclassified controls and moderate and severe Parkinson's disease (PD) participants.

| | | Controls | | | PD | | |
|---|---|---|---|---|---|---|---|
| | | False positive | True negative | $p$ | False negative | True positive | $p$ |
| Moderate PD | | | | | | | |
| | UPDRS_PIII | – | – | – | 23.2 ± 13.2 | 28.8 ± 9.8 | 0.20 |
| | MoCA | – | – | – | 27.4 ± 2.3 | 27.2 ± 3.1 | 0.85 |
| | CIRS-G | – | – | – | 5.3 ± 3.7 | 5.7 ± 3.9 | 0.84 |
| | Age | 63.8 ± 12.0 | 64.2 ± 12.0 | 0.52 | 65.4 ± 7.4 | 69.8 ± 8.7 | 0.13 |
| | Sex (% male) | 16.7 | 40.1 | 0.28 | 80.0 | 69.1 | 0.63 |
| | Medication state (% ON) | – | – | – | 100 | 67.3 | 0.34 |
| | H&Y (n) | | | – | | | 0.72 |
| | 2.5 | | | | 4 | 31 | |
| | 3 | | | | 1 | 24 | |
| Severe PD | | | | | | | |
| | UPDRS_PIII | – | – | – | 19.5 ± 9.2 | 42.9 ± 9.5 | 0.07 |
| | MoCA | – | – | – | 29.5 ± 0.71 | 24.9 ± 5.0 | 0.005 |
| | CIRS-G | – | – | – | 5.0 ± 2.8 | 7.5 ± 3.8 | 0.40 |
| | Age | – | – | – | 65.0 ± 15.6 | 74.4 ± 7.8 | 0.27 |
| | Sex (% male) | – | – | – | 50.0 | 66.7 | 0.66 |
| | Medication state (% ON) | – | – | – | 100 | 100 | – |
| | H&Y (n) | | | – | | | – |
| | 4 | | | | 2 | 15 | |

Following the same simplification strategies applied to all PD and mild PD classification, the effectiveness of the automated segmentation and the redundancy of complex calculations of kinesiological features were once more demonstrated through a comparison between our standard models, which used data from segmented tasks and excluded kinesiological features to alternative models. These alternative models either used features derived from data of the unsegmented tasks or included the kinesiological features in addition to the time- and frequency-domain features from the algorithmic segmented tasks. Models employing data from unsegmented tasks reduced the accuracy by 4.5% for moderate classification and had similar performance for severe PD classification (Supplementary Table S9, 2nd column vs 1st column). Similarly, models incorporating kinesiological features dropped the accuracy by 2.7% and 1.5% compared to our standard models for moderate and severe PD classification, respectively (Supplementary Table S9, 3rd column vs 1st column).

**Table S9.** Accuracy (%) of super learner models using the unsegmented tasks and combination of segmented tasks and calculated kinesiological features to distinguish controls from PD participants. The numbers in parentheses represent the difference in accuracy between each model and the corresponding standard model, which used data from segmented tasks without incorporating kinesiological features (same row).

| | With segmented tasks and no kinesiological features | With unsegmented tasks and no kinesiological features | With segmented tasks and kinesiological features |
|---|---|---|---|
| Moderate PD (H&Y 2.5, 3) vs controls | 90.0 | 85.5 (-4.5) | 87.3 (-2.7) |
| Severe PD (H&Y 4) vs controls | 97.0 | 97.0 (0.0) | 95.5 (-1.5) |

Additionally, we compared the performance of models based solely on TUG or cogTUG features to our standard models incorporating features from all segmented mobility tasks (see Supplementary Table S5 for the selected features). We observed that cogTUG-only models yielded better results than TUG-only models and increased the accuracy by 2.7% compared to the standard models for the moderate stages of the disease (Supplementary Table S10,

columns 2, 3). In severe stages, both the TUG-only and cogTUG-only models showed the same performance, with a slight decrease in accuracy (1.5%) compared to the standard model (Supplementary Table S10, columns 2, 3). In summary, these findings demonstrate again that sensor data from a single, more complex mobility task incorporating multiple movements may achieve satisfactory performance in distinguishing PD and control participants.

**Table S10.** Accuracy (%) of alternative models. TUG-only and cogTUG-only models used features derived from the first trial, the second trial, and the mean of corresponding features from both trials. TUG-duration and cogTUG-duration models used the duration of TUG and cogTUG tasks. The numbers in parentheses represent the difference in accuracy between the respective model and the corresponding all-tasks model (same row).

| | All tasks | TUG-only | cogTUG-only | TUG-duration | cogTUG-duration |
|---|---|---|---|---|---|
| Moderate PD (H&Y 2.5, 3) vs controls | 90.0 | 81.8 (-8.2) | 92.7 (+2.7) | 79.1 (-10.9) | 82.7 (-7.3) |
| Severe PD (H&Y 4) vs controls | 97.0 | 95.5 (-1.5) | 95.5 (-1.5) | 94.0 (-3.0) | 92.5 (-4.5) |

Furthermore, the possibility of achieving further simplification without significantly compromising model performance was examined by assessing whether total duration of TUG and cogTUG could serve as a single feature sufficient to construct a classifier with comparable predictive performance. Consistent with the results for the all PD and mild PD classification, participants at moderate and severe PD stages exhibited significantly longer durations than control participants. However, the constructed logistic regression models using total duration as the only predictor decreased the accuracy in distinguishing PD from control participants by at least 3.0% compared to other more complex and comprehensive models (Supplementary Table S10, columns 4, 5). Once more, TUG/cogTUG duration alone did not reflect the discriminatory potential of these complex tasks.

## S2.2 Feature-level importance

SHAP values in Supplementary Figure S4 show that low values of mean binarized values of the second turn, variation fractal dimension of the first turn, frequency at the first peak of Discrete Fourier Transform (DFT) of stand-to-sit transition, and minimum value of first walk features derived from the cogTUG task increased the probability of belonging to the PD class. Inversely, high values of slope sign change of stand-to-sit and mean absolute value slope of stand-to-sit, which were calculated from the cogTUG task, and waveform length derived from the standing with eyes closed increased the probability of belonging to the PD class.

In Supplementary Figure S6, using SHAP values of the cogTUG features we found that low values of variation fractal dimension of the first turn, mean binarized values of the second turn, the frequency at the third peak of DFT of the second turn, and the frequency at the first peak of DFT of stand-to-sit increased the probability of belonging to the PD class. Whereas, high values of the total power of the first walk and skewness of DFT power of stand-to-sit increased the probability of belonging to the PD class. Similarly, high values of the DFT frequency ratio for the standing with eyes closed task increased the probability of being classified as PD.

Supplementary Figures S9 and S10 show that scores from the Older Americans Resource Survey-Instrumental Activities of Daily Living (OARS) questionnaire were from among the selected by our models for classifying later stages of the disease. OARS comprise 14 questions: 7 activities of daily living (ADLs); and 7 instrumental activities of daily living (IADLs). ADLs are essential for self-care (walking, eating, bathing, dressing, grooming, getting in/out of bed, and using the toilet) and IADLs are necessary to function independently in the environment (housework, shopping, traveling, preparing meals, using the telephone, managing medication, and handling money). Each question has five possible responses: no difficulty, slower/with greater difficulty, needs some help, needs moderate help, or is completely unable. Higher scores indicate increased disability. Total OARS, ADL, and IADL scores were important in identifying moderate PD participants, and the total OARS score was important in identifying severe PD participants. SHAP values show that low values of the frequency at the fifth peak of Lomb-Scargle Periodogram (LSP) of sit-to-stand, and DFT mean power of stand-to-sit features increased the probability of belonging to the PD class. Whereas, high values of slope sign change of stand-to-sit, the simple square interval of the second walk, integrated absolute values of first walk and stand-to-sit cogTUG features, integrated absolute values of the second walk from the second TUG trial, and total OARS score features increased the probability of being classified as PD.

## S2.3 Single feature-based models: total TUG/cogTUG duration

We compared the total duration of TUG and cogTUG using two-tailed Student's t-test. The analysis revealed a significant time difference between PD and control (ctrl) participants (TUG $ctrl = 11.7 \pm 2.3$ $s.d.$, $PD = 15.6 \pm 6.4$ $s.d.$, $p = 1.3 \times 10^{-12}$; cogTUG $ctrl = 14.4 \pm 3.4$ $s.d.$, $PD = 21.3 \pm 9.3$ $s.d.$, $p = 2.2 \times 10^{-16}$, see Supplementary Figure S14, rows 1, 2). Comparison of subtask durations within TUG and cogTUG shows significant differences between PD and control groups, and that these differences are distributed among all subtasks (Supplementary Tables S11 and S12, top row).

Total duration of the task (in seconds) increased with PD severity (TUG mild PD $= 14.1 \pm 3.1$ $s.d.$, moderate PD $= 16.6 \pm 4.6$ $s.d.$, severe PD $= 27.8 \pm 17.2$ $s.d.$), and with adding the cognitive test element to the movement task (cogTUG mild PD $= 19.1 \pm 6.3$ $s.d.$, moderate PD $= 23.6 \pm 7.0$ $s.d.$, severe PD $= 38.0 \pm 19.5$ $s.d.$) (Supplementary Figure S14, rows 3-5). Similar patterns were observed for the duration of each subtask (Supplementary Tables S11 and S12, rows 2-4). In comparison to the control participants, participants at different PD stages exhibited significantly longer durations when compared using two-tailed Student's t-test ($p < 0.0014$).

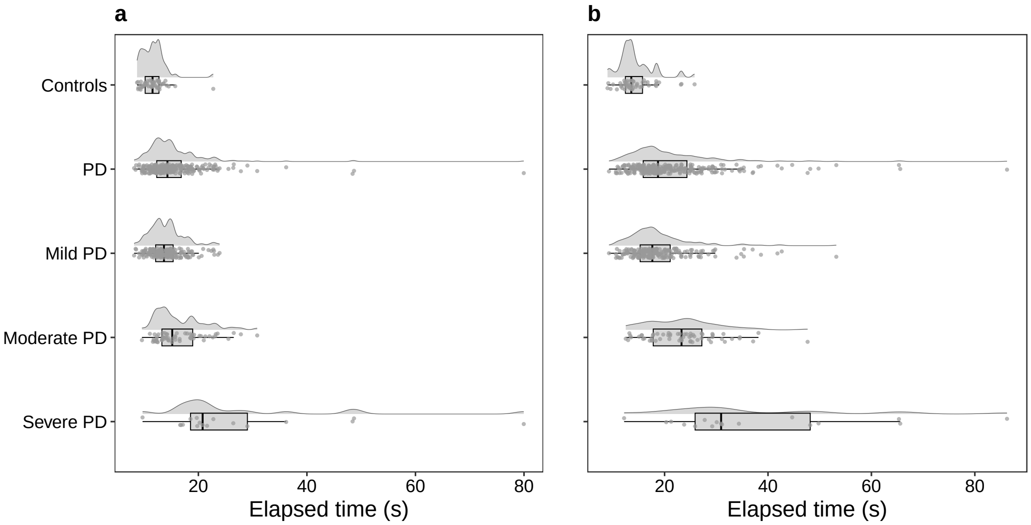


**Figure S14.** Comparison of the total duration of **(a)** TUG and **(b)** cogTUG tasks between different groups of PD participants and controls. Different PD groups shows significantly longer durations when compared to the controls using two-tailed Student's t-test ($p < 0.0014$).

**Table S11.** Comparing the duration of TUG sub-tasks between different groups of PD participants and controls (ctrl).

| | sit-to-stand | stand-to-sit | first turn | second turn | first walk | second walk |
|---|---|---|---|---|---|---|
| PD vs controls | PD: 1.9 ± 0.93<br>ctrl: 1.5 ± 0.34<br>$p = 8.4 \times 10^{-8}$ | PD: 3.4 ± 2.2<br>ctrl: 2.4 ± 0.68<br>$p = 2.2 \times 10^{-9}$ | PD: 1.8 ± 0.61<br>ctrl: 1.5 ± 0.35<br>$p = 7.0 \times 10^{-8}$ | PD: 1.8 ± 0.72<br>ctrl: 1.3 ± 0.31<br>$p = 2.7 \times 10^{-10}$ | PD: 3.1 ±1.5<br>ctrl: 2.5 ± 0.63<br>$p = 5.1 \times 10^{-6}$ | PD: 3.2 ± 2.0<br>ctrl: 2.5 ± 0.82<br>$p = 3.2 \times 10^{-5}$ |
| Mild PD vs controls | PD: 1.8 ± 0.61<br>ctrl: 1.5 ± 0.34<br>$p = 0.0002$ | PD: 3.0 ± 1.1<br>ctrl: 2.4 ± 0.68<br>$p = 3.3 \times 10^{-7}$ | PD: 1.7 ± 0.46<br>ctrl: 1.5 ± 0.35<br>$p = 6.5 \times 10^{-4}$ | PD: 1.6 ± 0.48<br>ctrl: 1.3 ± 0.31<br>$p = 7.7 \times 10^{-7}$ | PD: 2.8 ± 0.83<br>ctrl: 2.5 ± 0.63<br>$p = 0.04$ | PD: 2.8 ± 0.89<br>ctrl: 2.5 ± 0.82<br>$p = 0.013$ |
| Moderate PD vs controls | PD: 2.1 ± 1.1<br>ctrl: 1.5 ± 0.34<br>$p = 4.0 \times 10^{-5}$ | PD: 3.6 ± 1.6<br>ctrl: 2.4 ± 0.68<br>$p = 3.9 \times 10^{-7}$ | PD: 2.0 ± 0.63<br>ctrl: 1.5 ± 0.35<br>$p = 6.3 \times 10^{-8}$ | PD: 1.8 ± 0.60<br>ctrl: 1.3 ± 0.31<br>$p = 1.2 \times 10^{-7}$ | PD: 3.6 ± 1.5<br>ctrl: 2.5 ± 0.63<br>$p = 1.6 \times 10^{-6}$ | PD: 3.5 ± 1.1<br>ctrl: 2.5 ± 0.82<br>$p = 2.9 \times 10^{-7}$ |
| Severe PD vs controls | PD: 2.9 ± 2.0<br>ctrl: 1.5 ± 0.34<br>$p = 0.011$ | PD: 6.7 ± 6.7<br>ctrl: 2.4 ± 0.68<br>$p = 0.017$ | PD: 2.7 ± 0.97<br>ctrl: 1.5 ± 0.35<br>$p = 8.0 \times 10^{-5}$ | PD: 2.8 ± 1.8<br>ctrl: 1.3 ± 0.31<br>$p = 0.004$ | PD: 5.6 ± 3.5<br>ctrl: 2.5 ± 0.63<br>$p = 0.002$ | PD: 6.6 ± 6.5<br>ctrl: 2.5 ± 0.82<br>$p = 0.019$ |

**Table S12.** Comparing the duration of cogTUG sub-tasks between different groups of PD participants and controls (ctrl).

| | sit-to-stand | stand-to-sit | first turn | second turn | first walk | second walk |
|---|---|---|---|---|---|---|
| PD vs controls | PD: 2.8 ± 1.6<br>ctrl: 1.9 ± 0.50<br>$p = 1.2 \times 10^{-12}$ | PD: 4.0 ± 2.0<br>ctrl: 2.5 ± 0.73<br>$p = 3.4 \times 10^{-16}$ | PD: 2.2 ± 0.90<br>ctrl: 1.6 ± 0.37<br>$p = 2.1 \times 10^{-13}$ | PD: 2.2 ± 1.0<br>ctrl: 1.5 ± 0.44<br>$p = 9.2 \times 10^{-12}$ | PD: 4.6 ± 3.8<br>ctrl: 3.2 ± 1.2<br>$p = 7.0 \times 10^{-7}$ | PD: 5.0 ± 2.8<br>ctrl: 3.6 ± 1.3<br>$p = 1.3 \times 10^{-7}$ |
| Mild PD vs controls | PD: 2.5 ± 1.1<br>ctrl: 1.9 ± 0.50<br>$p = 1.5 \times 10^{-8}$ | PD: 3.6 ± 1.3<br>ctrl: 2.5 ± 0.73<br>$p = 2.5 \times 10^{-12}$ | PD: 2.0 ± 0.62<br>ctrl: 1.6 ± 0.37<br>$p = 3.0 \times 10^{-9}$ | PD: 2.0 ± 0.73<br>ctrl: 1.5 ± 0.44<br>$p = 4.3 \times 10^{-8}$ | PD: 4.1 ± 3.3<br>ctrl: 3.2 ± 1.2<br>$p = 0.003$ | PD: 4.3 ± 2.1<br>ctrl: 3.6 ± 1.3<br>$p = 0.0018$ |
| Moderate PD vs controls | PD: 3.2 ± 1.3<br>ctrl: 1.9 ± 0.50<br>$p = 6.2 \times 10^{-10}$ | PD: 4.2 ± 1.4<br>ctrl: 2.5 ± 0.73<br>$p = 1.6 \times 10^{-11}$ | PD: 2.4 ± 1.0<br>ctrl: 1.6 ± 0.37<br>$p = 2.9 \times 10^{-7}$ | PD: 2.4 ± 1.0<br>ctrl: 1.5 ± 0.44<br>$p = 7.1 \times 10^{-8}$ | PD: 5.1 ± 3.4<br>ctrl: 3.2 ± 1.2<br>$p = 1.2 \times 10^{-4}$ | PD: 5.9 ± 2.9<br>ctrl: 3.6 ± 1.3<br>$p = 2.1 \times 10^{-7}$ |
| Severe PD vs controls | PD: 4.3 ± 4.1<br>ctrl: 1.9 ± 0.50<br>$p = 0.025$ | PD: 6.9 ± 5.2<br>ctrl: 2.5 ± 0.73<br>$p = 0.003$ | PD: 3.2 ± 1.8<br>ctrl: 1.6 ± 0.37<br>$p = 0.0013$ | PD: 3.2 ± 2.2<br>ctrl: 1.5 ± 0.44<br>$p = 0.0049$ | PD: 9.4 ± 6.4<br>ctrl: 3.2 ± 1.2<br>$p = 0.001$ | PD: 8.4 ± 5.0<br>ctrl: 3.6 ± 1.3<br>$p = 1.0 \times 10^{-3}$ |

# S3 Supplementary Files

The supplementary material available at https://doi.org/10.13016/dspace/ddzp-im5e contains the following compressed files:

1. **sensor_data**: folder with sensor readings derived from 32-foot walk, standing with eyes open, standing with eyes closed, two trials of TUG, and two trials of cogTUG. It has also the calculated kinesiological variables, demographics, and clinical evaluation data in separate files.

2. **code_notebook**: notebook with the code used to generate the super learner models. It has sections corresponding to the components of the proposed machine-learning pipeline. To view the notebook open the file `index.html` in a web browser or open the file `notebook.pdf`.

3. **rdata**: folder with intermediate R objects.

    `sensor_features.RData`: saves a list of the features table of each task.

    `sensor_features_all_tasks.RData`: saves one table of features for all subjects not missing cogTUG data and tasks. The mean of repeated tasks and demographics variables are also added.

    `PD_control_seg.RData`: save a data frame with rows corresponding to PD participants and controls and columns corresponding to the features selected by the feature reduction technique.

    `HY_control_early.RData`: save a data frame with rows corresponding to mild PD participants and controls and columns corresponding to the features selected by the feature reduction technique.

    `HY_control_mild.RData`: save a data frame with rows corresponding to moderate PD participants and controls and columns corresponding to the features selected by the feature reduction technique.

    `HY_control_severe.RData`: save a data frame with rows corresponding to severe PD participants and controls and columns corresponding to the features selected by the feature reduction technique.

    `var_reduct_PD_control_splits.RData` saves the training and test splits for the five repeats and five-fold cross-validation framework used to build a classifier distinguishing PD patients and controls.

    `var_reduct_HY_early_HC_splits.RData` saves the training and test splits for the five repeats and five-fold cross-validation framework used to build a classifier distinguishing mild PD participants and controls.

    `var_reduct_HY_mild_HC_splits.RData` saves the training and test splits for the five repeats and five-fold cross-validation framework used to build a classifier distinguishing moderate PD participants and controls.

    `var_reduct_HY_severe_HC_splits.RData` saves the training and test splits for the five repeats and five-fold cross-validation framework used to build a classifier distinguishing severe PD participants and controls.

4. **files**: for each classifier, its folder contains five `sl_predictions.csv` files with the predictions of the super learner models for the five repeats of the outer loop, `train_test_files` with the train and test split files, `top_imp_scores.csv` with the permutation-based importance scores, and `top_shap_values.csv` with the SHAP values of each feature. Each classifier folder has also five files (`GLM_params.csv`, `GBM_params.csv`, `DRF_params`, `XGBoost_params.csv`, `DeepLearning_params.csv`) with the hyperparameters of the base models used to build the super learners.

5. **models**: for each classifier, it has the 25 `superlearner` models built inside the nested loop framework.

6. **README**: file with detailed instructions on how to set up and run the code, as well as any dependencies or requirements.

# References


1. Schlachetzki, J.C.; Barth, J.; Marxreiter, F.; Gossler, J.; Kohl, Z.; Reinfelder, S.; Gassner, H.; Aminian, K.; Eskofier, B.M.; Winkler, J.; et al. Wearable sensors objectively measure gait parameters in Parkinson's disease. *PLOS ONE* **2017**, 12, 1–18.

2. Krupicka, R.; Viteckova, S.; Cejka, V.; Klempir, O.; Szabo, Z.; Ruzicka, E. Bradykan: a motion capture system for objectification of hand motor tests in Parkinson disease. In *2017 E-Health and Bioengineering Conference (EHB)* **2017** , pp. 446–449.

3. Abtahi, M.; Bahram Borgheai, S.; Jafari, R.; Constant, N.; Diouf, R.; Shahriari, Y.; Mankodiya, K. Merging fNIRS-EEG brain monitoring and body motion capture to distinguish Parkinson's disease. *IEEE Trans. Neural Syst. Rehabil. Eng.* **2020**, 28(6), 1246–1253.

4. Castaño-Pino, Y.J.; González, M.C.; Quintana-Peña, V.; Valderrama, J.; Muñoz, B.; Orozco, J.; Navarro, A. Automatic gait phases detection in Parkinson disease: a comparative study. In *2020 42nd Annual International Conference of the IEEE Engineering in Medicine Biology Society (EMBC)* **2020** , pp. 798–802.

5. Phan, D.; Horne, M.; Pathirana, P.N.; Farzanehfar, P. Measurement of axial rigidity and postural instability using wearable sensors. *Sensors* **2018**, 18(2), 495.

6. Del Din, S.; Elshehabi, M.; Galna, B.; Hobert, M.A.; Warmerdam, E.; Suenkel, U.; Brockmann, K.; Metzger, F.; Hansen, C.; Berg, D.; et al. Gait analysis with wearables predicts conversion to Parkinson disease. *Ann. Neurol.* **2019**, 86(3), 357–367.

7. Rovini, E.; Maremmani, C.; Moschetti, A.; Esposito, D.; Cavallo, F. Comparative motor pre-clinical assessment in Parkinson's disease using supervised machine learning approaches. *Ann. Biomed. Eng.* **2018**, 46, 2057–2068.

8. Rehman, R.Z.U.; Del Din, S.; Shi, J.Q.; Galna, B.; Lord, S.; Yarnall, A.J.; Guan, Y.; Rochester, L. Comparison of walking protocols and gait assessment systems for machine learning-based classification of Parkinson's disease. *Sensors* **2019**, 19(24), 5363.

9. Buckley, C.; Galna, B.; Rochester, L.; Mazzà, C. Quantification of upper body movements during gait in older adults and in those with Parkinson's disease: impact of acceleration realignment methodologies. *Gait Posture* **2017**, 52, 265–271.

10. Cai, G.; Shi, W.; Wang, Y.; Weng, H.; Chen, L.; Yu, J.; Chen, Z.; Lin, F.; Ren, K.; Zeng, Y.; et al. Specific distribution of digital gait biomarkers in Parkinson's disease using body-worn sensors and machine learning. *J. Gerontol. A, Biol. Sci. Med. Sci.* **2023**, 78(8), 1348–1354.

11. Jovanovic, L.; Damaševičius, R.; Matić, R.; Kabiljo, M.; Simic, V.; Kunjadic, G.; Antonijevic, M.; Zivkovic, M.; Bacanin, N. Detecting Parkinson's disease from shoe-mounted accelerometer sensors using convolutional neural networks optimized with modified metaheuristics. *PeerJ Comput. Sci.* **2024**, 10, e2031.

12. Battista, L.; Romaniello, A. A new wrist-worn tool supporting the diagnosis of parkinsonian motor syndromes. *Sensors* **2024**, 24(6), 1965.

13. Yue, P.; Li, Z.; Zhou, M.; Wang, X.; Yang, P. Wearable-sensor-based weakly supervised Parkinson's disease assessment with data augmentation. *Sensors* **2024**, 24(4), 1196.

14. Bailo, G.; Saibene, F.L.; Bandini, V.; Arcuri, P.; Salvatore, A.; Meloni, M.; Castagna, A.; Navarro, J.; Lencioni, T.; Ferrarin, M.; et al. Characterization of walking in mild Parkinson's disease: reliability, validity and discriminant ability of the six-minute walk test instrumented with a single inertial sensor. *Sensors* **2024**, 24(2), 662.

15. Nair, P.; Baghini, M.S.; Pendharkar, G.; Chung, H. Detecting early-stage Parkinson's disease from gait data. *Proc. Inst. Mech. Eng. H* **2023**, 237(11), 1287–1296.

16. Keloth, S.M.; Viswanathan, R.; Jelfs, B.; Arjunan, S.; Raghav, S.; Kumar, D. Which gait parameters and walking patterns show the significant differences between Parkinson's disease and healthy participants? *Biosensors* **2019**, 9(2), 59.

17. Trabassi, D.; Serrao, M.; Varrecchia, T.; Ranavolo, A.; Coppola, G.; De Icco, R.; Tassorelli, C.; Castiglia, S.F. Machine learning approach to support the detection of Parkinson's disease in IMU-based gait analysis. *Sensors* **2022**, 22(10), 3700.

18. Marin, F.; Warmerdam, E.; Marin, Z.; Ben Mansour, K.; Maetzler, W.; Hansen, C. Scoring the sit-to-stand performance of Parkinson's patients with a single wearable sensor. *Sensors* **2022**, 22(21), 8340.

19. Gourrame, K.; Griškevičius, J.; Haritopoulos, M.; Lukšys, D.; Jatužis, D.; Kaladytė-Lokominienė, R.; Bunevičiūtė, R.; Mickutė, G. Parkinson's disease classification with CWNN: using wavelet transformations and IMU data fusion for improved accuracy. *Technol. Health Care* **2023**, 31(6), 2447–2455.

20. Meng, L.; Pang, J.; Yang, Y.; Chen, L.; Xu, R.; Ming, D. Inertial-based gait metrics during turning improve the detection of early-stage Parkinson's disease patients. *IEEE Trans. Neural Syst. Rehabil. Eng.* **2023**, 31, 1472–1482.

21. Caramia, C.; Torricelli, D.; Schmid, M.; Muñoz-Gonzalez, A.; Gonzalez-Vargas, J.; Grandas, F.; Pons, J.L. IMU-based classification of Parkinson's disease from gait: a sensitivity analysis on sensor location and feature selection. *IEEE J. Biomed. Health Inform.* **2018**, 22(6), 1765–1774.

22. Mahadevan, N.; Demanuele, C.; Zhang, H.; Volfson, D.; Ho, B.; Erb, M.; Patel, S. Development of digital biomarkers for resting tremor and bradykinesia using a wrist-worn wearable device. *npj Digit. Med.* **2020**, 3, 5.

23. Lin, Z.; Dai, H.; Xiong, Y.; Xia, X.; Horng, S.J. Quantification assessment of bradykinesia in Parkinson's disease based on a wearable device. In *2017 39th Annual International Conference of the IEEE Engineering in Medicine and Biology Society (EMBC)* **2017** , pp. 803–806.

24. Borzì, L.; Mazzetta, I.; Zampogna, A.; Suppa, A.; Irrera, F.; Olmo, G. Predicting axial impairment in Parkinson's disease through a single inertial sensor. *Sensors* **2022**, 22(2), 412.

25. Ymeri, G.; Salvi, D.; Olsson, C.M.; Wassenburg, M.V.; Tsanas, A.; Svenningsson, P. Quantifying Parkinson's disease severity using mobile wearable devices and machine learning: the ParkApp pilot study protocol. *BMJ Open* **2023**, 13(12).

26. Bobić, V.; Djurić-Jovičić, M.; Dragašević, N.; Popović, M.B.; Kostić, V.S.; Kvaščev, G. An expert system for quantification of bradykinesia based on wearable inertial sensors. *Sensors* **2019**, 19(11), 2644.

27. Han, Y.; Liu, X.; Zhang, N.; Zhang, X.; Zhang, B.; Wang, S.; Liu, T.; Yi, J. Automatic assessments of parkinsonian gait with wearable sensors for human assistive systems. *Sensors* **2023**, 23(4), 2104.

28. Heijmans, M.; Habets, J.; Herff, C.; Aarts, J.; Stevens, A.; Kuijf, M.; Kubben, P. Monitoring Parkinson's disease symptoms during daily life: a feasibility study. *npj Park. Dis.* **2019**, 5, 21.

29. Wu, Z.; Jiang, X.; Zhong, M.; Shen, B.; Zhu, J.; Pan, Y.; Dong, J.; Xu, P.; Zhang, W.; Zhang, l. Wearable sensors measure ankle joint changes of patients with Parkinson's disease before and after acute levodopa challenge. *Park. Dis.* **2020**, 2020, 2976535.

30. Wu, X.; Ma, L.; Wei, P.; Shan, Y.; Chan, P.; Wang, K.; Zhao, G. Wearable sensor devices can automatically identify the ON-OFF status of patients with Parkinson's disease through an interpretable machine learning model. *Front. Neurol.* **2024**, 15, 1387477.

31. Pugh, R.J.; Higgins, R.D.; Min, H.; Wutzke, C.J.; Guccione, A.A. Turns while walking among individuals with Parkinson's disease following overground locomotor training: a pilot study. *Clin. Biomech.* **2024**, 114, 106234.

32. Sotirakis, C.; Su, Z.; Brzezicki, M.A.; Conway, N.; Tarassenko, L.; FitzGerald, J.J.; Antoniades, C.A. Identification of motor progression in Parkinson's disease using wearable sensors and machine learning. *npj Park. Dis.* **2023**, 9, 1–8.

33. Delrobaei, M.; Memar, S.; Pieterman, M.; Stratton, T.W.; McIsaac, K.; Jog, M. Towards remote monitoring of Parkinson's disease tremor using wearable motion capture systems. *J. Neurol. Sci.* **2018**, 384, 38–45.

34. Memar, S.; Delrobaei, M.; Pieterman, M.; McIsaac, K.; Jog, M. Quantification of whole-body bradykinesia in Parkinson's disease participants using multiple inertial sensors. *J. Neurol. Sci.* **2018**, 387, 157–165.

35. di Biase, L.; Summa, S.; Tosi, J.; Taffoni, F.; Marano, M.; Cascio Rizzo, A.; Vecchio, F.; Formica, D.; Di Lazzaro, V.; Di Pino, G.; et al. Quantitative analysis of bradykinesia and rigidity in Parkinson's disease. *Front. Neurol.* **2018**, 9, 121.

36. Delrobaei, M.; Baktash, N.; Gilmore, G.; McIsaac, K.; Jog, M. Using wearable technology to generate objective Parkinson's disease dyskinesia severity score: possibilities for home monitoring. *IEEE Trans. Neural Syst. Rehabil. Eng.* **2017**, 25(10), 1853–1863.

37. Singh, M.; Prakash, P.; Kaur, R.; Sowers, R.; Brašić, J.R.; Hernandez, M.E. A deep learning approach for automatic and objective grading of the motor impairment severity in Parkinson's disease for use in tele-assessments. *Sensors* **2023**, 23(21), 9004.

38. Prakash, P.; Kaur, R.; Levy, J.; Sowers, R.; Brašić, J.; Hernandez, M.E. A deep learning approach for grading of motor impairment severity in Parkinson's disease. In *2023 45th Annual International Conference of the IEEE Engineering in Medicine and Biology Society (EMBC)* **2023** , pp. 1–4.

39. Bremm, R.P.; Pavelka, L.; Garcia, M.M.; Mombaerts, L.; Krüger, R.; Hertel, F. Sensor-based quantification of MDS-UPDRS III subitems in Parkinson's disease using machine learning. *Sensors* **2024**, 24(7), 2195.

40. Huo, W.; Angeles, P.; Tai, Y.; Pavese, N.; Wilson, S.; Hu, M.; Vaidyanathan, R. A heterogeneous sensing suite for multisymptom quantification of Parkinson's disease. *IEEE Trans. Neural Syst. Rehabil. Eng.* **2020**, 28(6), 1397–1406.

41. Yan, F.; Gong, J.; Zhang, Q.; He, H. Learning motion primitives for the quantification and diagnosis of mobility deficits. *IEEE Trans. Biomed. Eng.* **2024** , pp. 1–10.

42. Hssayeni, M.D.; Jimenez-Shahed, J.; Burack, M.A.; Ghoraani, B. Wearable sensors for estimation of parkinsonian tremor severity during free body movements. *Sensors* **2019**, 19(19), 4215.

43. Zajki-Zechmeister, T.; Kögl, M.; Kalsberger, K.; Franthal, S.; Homayoon, N.; Katschnig-Winter, P.; Wenzel, K.; Zajki-Zechmeister, L.; Schwingenschuh, P. Quantification of tremor severity with a mobile tremor pen. *Heliyon* **2020**, 6(8), e04702.

44. Lonini, L.; Dai, A.; Shawen, N.; Simuni, T.; Poon, C.; Shimanovich, L.; Daeschler, M.; Ghaffari, R.; Rogers, J.; Jayaraman, A. Wearable sensors for Parkinson's disease: which data are worth collecting for training symptom detection models. *npj Digit. Med.* **2018**, 1, 64.

45. Tsakanikas, V.; Ntanis, A.; Rigas, G.; Androutsos, C.; Boucharas, D.; Tachos, N.; Skaramagkas, V.; Chatzaki, C.; Kefalopoulou, Z.; Tsiknakis, M.; et al. Evaluating gait impairment in Parkinson's disease from instrumented insole and IMU sensor data. *Sensors* **2023**, 23(8), 3902.

46. Chomiak, T.; Xian, W.; Pei, Z.; Hu, B. A novel single-sensor-based method for the detection of gait-cycle breakdown and freezing of gait in Parkinson's disease. *J. Neural Transm.* **2019**, 126, 1029–1036.

47. Punin, C.; Barzallo, B.; Clotet, R.; Bermeo, A.; Bravo, M.; Bermeo, J.P.; Llumiguano, C. A non-invasive medical device for Parkinson's patients with episodes of freezing of gait. *Sensors* **2019**, 19(3), 737.

48. Li, B.; Zhang, Y.; Tang, L.; Gao, C.; Gu, D. Automatic detection system for freezing of gait in Parkinson's disease based on the clustering algorithm. In *2018 2nd IEEE advanced information management, communicates, electronic and automation control conference (IMCEC)* **2018** , pp. 1640–4.

49. Camps, J.; Samà, A.; Martín, M.; Rodríguez-Martín, D.; Pérez-López, C.; Moreno Arostegui, J.M.; Cabestany, J.; Català, A.; Alcaine, S.; Mestre, B.; et al. Deep learning for freezing of gait detection in Parkinson's disease patients in their homes using a waist-worn inertial measurement unit. *Knowl. -Based Syst.* **2018**, 139, 119–131.

50. Samà, A.; Rodríguez-Martín, D.; Pérez-López, C.; Català, A.; Alcaine, S.; Mestre, B.; Prats, A.; Crespo, M.C.; Bayés, À. Determining the optimal features in freezing of gait detection through a single waist accelerometer in home environments. *Pattern Recognit. Lett.* **2018**, 105, 135–143.

51. Capecci, M.; Pepa, L.; Verdini, F.; Ceravolo, M.G. A smartphone-based architecture to detect and quantify freezing of gait in Parkinson's disease. *Gait Posture* **2016**, 50, 28–33.

52. Reches, T.; Dagan, M.; Herman, T.; Gazit, E.; Gouskova, N.A.; Giladi, N.; Manor, B.; Hausdorff, J.M. Using wearable sensors and machine learning to automatically detect freezing of gait during a FOG-provoking test. *Sensors* **2020**, 20(16), 4474.

53. Pham, T.T.; Moore, S.T.; Lewis, S.J.G.; Nguyen, D.N.; Dutkiewicz, E.; Fuglevand, A.J.; McEwan, A.L.; Leong, P.H. Freezing of gait detection in Parkinson's disease: a subject-independent detector using anomaly scores. *IEEE Trans. Biomed. Eng.* **2017**, 64(11), 2719–2728.

54. Masiala, S.; Huijbers, W.; Atzmueller, M. Feature-set-engineering for detecting freezing of gait in Parkinson's disease using deep recurrent neural networks. *arXiv* **2019**. Article 1909.03428.

55. Mancini, M.; Shah, V.; Stuart, S.; Curtze, C.; Horak, F.; Safarpour, D.; Nutt, J. Measuring freezing of gait during daily-life: an open-source, wearable sensors approach. *J. NeuroEng. Rehabil.* **2021**, 18(1), 1.

56. Marcante, A.; Di Marco, R.; Gentile, G.; Pellicano, C.; Assogna, F.; Pontieri, F.E.; Spalletta, G.; Macchiusi, L.; Gatsios, D.; Giannakis, A.; et al. Foot pressure wearable sensors for freezing of gait detection in Parkinson's disease. *Sensors* **2021**, 21(1), 128.

57. Sigcha, L.; Costa, N.; Pavón, I.; Costa, S.; Arezes, P.; López, J.M.; De Arcas, G. Deep learning approaches for detecting freezing of gait in Parkinson's disease patients through on-body acceleration sensors. *Sensors* **2020**, 20(7), 1895.

58. Bikias, T.; Iakovakis, D.; Hadjidimitriou, S.; Charisis, V.; Hadjileontiadis, L.J. DeepFoG: an IMU-based detection of freezing of gait episodes in Parkinson's disease patients via deep learning. *Front. Robotics AI* **2021**, 8.

59. Pardoel, S.; Shalin, G.; Nantel, J.; Lemaire, E.D.; Kofman, J. Early detection of freezing of gait during walking using inertial measurement unit and plantar pressure distribution data. *Sensors* **2021**, 21(6), 2246.

60. Shi, B.; Tay, A.; Au, W.L.; Tan, D.M.L.; Chia, N.S.Y.; Yen, S.C. Detection of freezing of gait using convolutional neural networks and data from lower limb motion sensors. *IEEE Trans. Biomed. Eng.* **2022**, 69(7), 2256–2267.

61. Rennie, L.; Löfgren, N.; Moe-Nilssen, R.; Opheim, A.; Dietrichs, E.; Franzén, E. The reliability of gait variability measures for individuals with Parkinson's disease and healthy older adults – the effect of gait speed. *Gait Posture* **2018**, 62, 505–509.

62. Myers, P.S.; McNeely, M.E.; Pickett, K.A.; Duncan, R.P.; Earhart, G.M. Effects of exercise on gait and motor imagery in people with Parkinson disease and freezing of gait. *Parkinsonism Relat. Disord.* **2018**, 53, 89–95.

63. Zadka, A.; Rabin, N.; Gazit, E.; Mirelman, A.; Nieuwboer, A.; Rochester, L.; Del Din, S.; Pelosin, E.; Avanzino, L.; Bloem, B.R.; et al. A wearable sensor and machine learning estimate step length in older adults and patients with neurological disorders. *npj Digit. Med.* **2024**, 7, 142.

64. Haji Ghassemi, N.; Hannink, J.; Roth, N.; Gaßner, H.; Marxreiter, F.; Klucken, J.; Eskofier, B.M. Turning analysis during standardized test using on-shoe wearable sensors in Parkinson's disease. *Sensors* **2019**, 19(14), 3103.

65. Nguyen, A.; Roth, N.; Haji Ghassemi, N.; Hannink, J.; Seel, T.; Klucken, J.; Gaßner, H.; Eskofier, B. Development and clinical validation of inertial sensor-based gait-clustering methods in Parkinson's disease. *J. NeuroEng. Rehabil.* **2019**, 16, 77.

66. Castelli Gattinara Di Zubiena, F.; Menna, G.; Mileti, I.; Zampogna, A.; Asci, F.; Paoloni, M.; Suppa, A.; Del Prete, Z.; Palermo, E. Machine learning and wearable sensors for the early detection of balance disorders in Parkinson's disease. *Sensors* **2022**, 22(24), 9903.

67. Liuzzi, P.; Carpinella, I.; Anastasi, D.; Gervasoni, E.; Lencioni, T.; Bertoni, R.; Carrozza, M.C.; Cattaneo, D.; Ferrarin, M.; Mannini, A. Machine learning based estimation of dynamic balance and gait adaptability in persons with neurological diseases using inertial sensors. *Sci. Rep.* **2023**, 13, 8640.

68. Stack, E.; Agarwal, V.; King, R.; Burnett, M.; Tahavori, F.; Janko, B.; Harwin, W.; Ashburn, A.; Kunkel, D. Identifying balance impairments in people with Parkinson's disease using video and wearable sensors. *Gait Posture* **2018**, 62, 321–326.

69. Jehu, D.; Nantel, J. Fallers with Parkinson's disease exhibit restrictive trunk control during walking. *Gait Posture* **2018**, 65, 246–250.

70. Zhang, X.; Jin, Y.; Wang, M.; Ji, C.; Chen, Z.; Fan, W.; Rainer, T.; Guan, Q.; Li, Q. The impact of anxiety on gait impairments in Parkinson's disease: insights from sensor-based gait analysis. *J. NeuroEng. Rehabil.* **2024**, 21, 68.

71. Romijnders, R.; Warmerdam, E.; Hansen, C.; Welzel, J.; Schmidt, G.; Maetzler, W. Validation of IMU-based gait event detection during curved walking and turning in older adults and Parkinson's disease patients. *J. Neuroeng. Rehabil.* **2021**, 18(1), 28.

72. Lukšys, D.; Griškevičius, J. Application of nonlinear analysis for the assessment of gait in patients with Parkinson's disease. *Technol. Health Care* **2022**, 30(1), 201–208.

73. Cooley, J.W.; Tukey, J.W. An algorithm for the machine calculation of complex fourier series. *Math. Comput.* **1965**, 19, 297–301.

74. Phinyomark, A.; Phukpattaranont, P.; Limsakul, C. Feature reduction and selection for EMG signal classification. *Expert Syst. Appl.* **2012**, 39(8), 7420–7431.

75. Altin, C.; Er, O. Comparison of different time and frequency domain feature extraction methods on elbow gesture's EMG. *Eur. J. Interdiscip. Stud.* **2016**, 5, 35.

76. Oung, Q.; Hariharan, M.; Lee, H.; Basah, S.; Sarillee, M.; Lee, C. Wearable multimodal sensors for evaluation of patients with Parkinson disease. In *2015 IEEE International Conference on Control System, Computing and Engineering (ICCSCE)* **2015** , pp. 269–274.

77. Sinderby, C.; Lindstrom, L.; Grassino, A.E. Automatic assessment of electromyogram quality. *J. Appl. Physiol.* **1995**, 79, 1803–1815.

78. Pepa, L.; Ciabattoni, L.; Verdini, F.; Capecci, M.; Ceravolo, M. Smartphone based fuzzy logic freezing of gait detection in Parkinson's disease. In *2014 IEEE/ASME 10th International Conference on Mechatronic and Embedded Systems and Applications (MESA)* **2014** , pp. 1–6.

79. Phinyomark, A.; Phukpattaranont, P.; Limsakul, C. Feature reduction and selection for EMG signal classification. *Expert Syst. Appl.* **2012**, 39, 7420–7431.

80. Mehta, A.; Vaddadi, S.K.; Sharma, V.; Kala, P. A phase-wise analysis of machine learning based human activity recognition using inertial sensors. In *2020 IEEE 17th India Council International Conference (INDICON)* **2020** , pp. 1–7.

81. Bao, L.; Intille, S.S. Activity recognition from user-annotated acceleration data. In Ferscha, A.; Mattern, F., editors, *Pervasive Computing*. Springer Berlin Heidelberg **2004** , pp. 1–17.

82. Arora, S.; Venkataraman, V.; Donohue, S.; Biglan, K.M.; Dorsey, E.R.; Little, M.A. High accuracy discrimination of Parkinson's disease participants from healthy controls using smartphones. In *2014 IEEE International Conference on Acoustics, Speech and Signal Processing (ICASSP)* **2014** , pp. 3641–3644.

83. Aich, S.; Youn, J.; Chakraborty, S.; Pradhan, P.M.; Park, J.H.; Park, S.; Park, J. A supervised machine learning approach to detect the on/off state in Parkinson's disease using wearable based gait signals. *Diagnostics* **2020**, 10, 421.

84. Phinyomark, A.; Phukpattaranont, P.; Limsakul, C. Fractal analysis features for weak and single-channel upper-limb EMG signals. *Expert Syst. Appl.* **2012**, 39(12), 11156–11163.

85. Hasni, H.; Yahya, N.; Asirvadam, V.S.; Jatoi, M.A. Analysis of electromyogram (EMG) for detection of neuromuscular disorders. In *2018 International Conference on Intelligent and Advanced System (ICIAS)* **2018** , pp. 1–6.

86. Sukumar, N.; Taran, S.; Bajaj, V. Physical actions classification of surface EMG signals using VMD. In *2018 International Conference on Communication and Signal Processing (ICCSP)* **2018** , pp. 0705–0709.

87. Kaiser, J. On a simple algorithm to calculate the 'energy' of a signal. In *International Conference on Acoustics, Speech, and Signal Processing* **1990** , pp. 381–384 vol.1.

88. Penzel, T.; Kantelhardt, J.; Grote, L.; Peter, J.; Bunde, A. Comparison of detrended fluctuation analysis and spectral analysis for heart rate variability in sleep and sleep apnea. *IEEE Trans. Biomed. Eng.* **2003**, 50(10), 1143–1151.

89. Higuchi, T. Approach to an irregular time series on the basis of the fractal theory. *Phys. D Nonlinear Phenom.* **1988**, 31, 277–283.

90. Katz, M.J. Fractals and the analysis of waveforms. *Comput. Biol. Med.* **1988**, 18(3), 145–156.

91. Gneiting, T.; Ševčíková, H.; Percival, D.B. Estimators of fractal dimension: assessing the roughness of time series and spatial data. *Stat. Sci.* **2012**, 27(2), 247–277.

92. Quiroz, J.C.; Banerjee, A.; Dascalu, S.M.; Lau, S.L. Feature selection for activity recognition from smartphone accelerometer data. *Intell. Autom. Soft Comput.* **2017** , pp. 1–9.

93. Ayman, A.; Attalah, O.; Shaban, H. An efficient human activity recognition framework based on wearable imu wrist sensors. In *2019 IEEE International Conference on Imaging Systems and Techniques (IST)* **2019** , pp. 1–5.

94. Batool, M.; Jalal, A.; Kim, K. Sensors technologies for human activity analysis based on svm optimized by pso algorithm. In *2019 International Conference on Applied and Engineering Mathematics (ICAEM)* **2019** , pp. 145–150.

95. Castiglia, S.F.; Trabassi, D.; Conte, C.; Ranavolo, A.; Coppola, G.; Sebastianelli, G.; Abagnale, C.; Barone, F.; Bighiani, F.; De Icco, R.; et al. Multiscale entropy algorithms to analyze complexity and variability of trunk accelerations time series in subjects with Parkinson's disease. *Sensors* **2023**, 23(10), 4983.

96. Venables, W.; Ripley, B. *Modern Applied Statistics with S.* Springer New York New York, NY **2002**.

97. Cover, T.M.; Thomas, J.A. *Elements of Information Theory.* John Wiley, New York **1990**.

98. Lundberg, S.M.; Lee, S.I. A unified approach to interpreting model predictions. In *Proceedings of the 31st International Conference on Neural Information Processing Systems* Red Hook, NY, USA **2017**. Curran Associates Inc. NIPS'17 , pp. 4768–4777.
